# PKM2 regulates metabolic flux and oxidative stress in the murine heart

**DOI:** 10.14814/phy2.70040

**Published:** 2024-09-10

**Authors:** Katie C. Y. Lee, Allison L. Williams, Lu Wang, Guoxiang Xie, Wei Jia, Anastasia Fujimoto, Mariana Gerschenson, Ralph V. Shohet

**Affiliations:** ^1^ Department of Medicine, John A. Burns School of Medicine University of Hawaii Honolulu Hawaii USA; ^2^ Department of Cell and Molecular Biology, John A. Burns School of Medicine University of Hawaii Honolulu Hawaii USA; ^3^ University of Hawaii Cancer Center Honolulu Hawaii USA

**Keywords:** glucose, glycolysis, metabolism, reactive oxygen species

## Abstract

Cardiac metabolism ensures a continuous ATP supply, primarily using fatty acids in a healthy state and favoring glucose in pathological conditions. Pyruvate kinase muscle (PKM) controls the final step of glycolysis, with PKM1 being the main isoform in the heart. PKM2, elevated in various heart diseases, has been suggested to play a protective role in cardiac stress, but its function in basal cardiac metabolism remains unclear. We examined hearts from global PKM2 knockout (PKM2^−/−^) mice and found reduced intracellular glucose. Isotopic tracing of U‐^13^C glucose revealed a shift to biosynthetic pathways in PKM2^−/−^ cardiomyocytes. Total ATP content was two‐thirds lower in PKM2^−/−^ hearts, and functional analysis indicated reduced mitochondrial oxygen consumption. Total reactive oxygen species (ROS) and mitochondrial superoxide were also increased in PKM2^−/−^ cardiomyocytes. Intriguingly, PKM2^−/−^ hearts had preserved ejection fraction compared to controls. Mechanistically, increased calcium/calmodulin‐dependent kinase II activity and phospholamban phosphorylation may contribute to higher sarcoendoplasmic reticulum calcium ATPase 2 pump activity in PKM2^−/−^ hearts. Loss of PKM2 led to altered glucose metabolism, diminished mitochondrial function, and increased ROS in cardiomyocytes. These data suggest that cardiac PKM2 acts as an important rheostat to maintain ATP levels while limiting oxidative stress. Although loss of PKM2 did not impair baseline contractility, its absence may make hearts more sensitive to environmental stress or injury.

## 1 INTRODUCTION

The heart requires large amounts of ATP to maintain contractility and provide blood flow to the body. Therefore, it has developed an adaptable and omnivorous metabolism, able to accept fatty acids, glucose, or ketone bodies as an energy source. The developing heart uses glucose and lactate as its main energy sources during embryogenesis and switches to the predominantly oxidative metabolism of fatty acids postpartum (Fisher et al., 1980). However, the heart can enhance glycolytic metabolism under stress, such as hypoxia (Kolwicz Jr. et al., 2013).

One of the mechanisms by which substrate utilization is optimized is alternative splicing of mRNAs (Baralle & Giudice, 2017). In the heart, pyruvate kinase muscle (PKM) is the final enzyme in glycolysis, catalyzing the conversion of phosphoenolpyruvate to pyruvate and generating ATP. Alternative splicing of *Pkm*, regulated by heterogeneous nuclear ribonucleoproteins (hnRNP) A1, A2, and I, produces two muscle isoforms in the heart: PKM1 and PKM2 (David et al., 2010). These splicing factors produce mutually exclusive incorporation of either exon 9 or 10 to generate PKM1 or PKM2, respectively. Both isoforms can form tetramers with high enzymatic activity that directs pyruvate to the Krebs cycle and oxidative metabolism (Israelsen & Vander Heiden, 2015). However, the balance of PKM2 dimer and tetramer can be allosterically regulated by numerous factors, including metabolites such as fructose‐1,6‐bisphosphate or posttranslational modifications such as phosphorylation of Y105 (Israelsen & Vander Heiden, 2015). The dimeric form exhibits reduced pyruvate kinase activity, promoting the conversion of pyruvate into lactate and enabling upstream glycolytic metabolites to enter the pentose phosphate pathway (PPP) (Israelsen & Vander Heiden, 2015). Recent evidence suggests that overexpression of PKM2 can redirect metabolites into the oxidative PPP and reduce oxidative damage in cardiomyocytes after myocardial infarction (MI) (Magadum et al., 2020).

PKM2 is highly expressed during embryogenesis and remains expressed at varying levels throughout the body in the adult (Israelsen & Vander Heiden, 2015). By contrast, PKM1 expression increases during maturation and differentiation and becomes the predominant isoform in the adult heart and other highly metabolic tissues (Israelsen & Vander Heiden, 2015). We have previously shown that ischemia‐induced alternative splicing of *Pkm* in the heart leads to increased PKM2. This is due in part to hypoxia‐inducible factor (HIF)‐1‐mediated increases in *Pkm2* transcript and its associated splicing factors (Williams et al., 2018). This switch in isoforms after MI was accompanied by an overall decrease in pyruvate kinase activity (Williams et al., 2018). PKM2 upregulation in the heart has also been observed in hypoxia, transverse aortic constriction (TAC)‐induced hypertrophy, heart failure, and other forms of cardiac injury (Lorenzana‐Carrillo et al., 2022; Ni et al., 2022; Rees et al., 2015). Aside from its metabolic activities, PKM2 overexpression has been found to attenuate cardiac hypertrophy and heart failure induced by pressure overload by phosphorylating the Rho family GTPase RAC1 (Ni et al., 2022). PKM2 has also been shown to have nuclear activity that enhances cardiomyocyte survival by stabilizing prosurvival transcription factors GATA4/6 and promoting the degradation of pro‐apoptotic p53 (Lorenzana‐Carrillo et al., 2022). Together, these studies suggest a cardioprotective role for PKM2 after injury.

While increased expression of PKM2 in disease settings may be beneficial, its role in basal cardiac metabolism is unclear. In this study, we found that PKM2 can regulate glucose metabolism in cardiomyocytes to influence the cellular oxidative state. PKM2 ablated hearts had high levels of reactive oxygen species (ROS) and reduced ATP content, indicative of mitochondrial and metabolic stress. These results indicate PKM2 may help maintain metabolic homeostasis, which may be particularly important during cardiac stress and injury.

## 2 METHODS

### 2.1 Mice and reagents

Approximately equal numbers of C57BL/6 male and female mice were used between 8 and 16 weeks of age (>25 g). All animal protocols and experiments were approved by the Institutional Animal Care and Use Committee of the University of Hawaii at Manoa (IACUC approval number 06–011‐17) and conform to the NIH Guide for the Care and Use of Laboratory Animals. PKM2^fl/fl^ (stock no. 024048) and CMV‐Cre (stock no. 006054) mouse lines were acquired from the Jackson Laboratory. Expression of Cre recombinase excised exon 10 (Pkm2) from PKM2^fl/fl^ mice, leading to nonsense‐mediated decay of Pkm2 transcripts lacking both exon 9 and 10 (Israelsen et al., 2013). Global Pkm2 knockout mice were then bred to remove the presence of Cre (hereafter called PKM2^−/−^). Mice were anesthetized using 5% isoflurane before euthanasia by CO_2_ and cervical dislocation. Antibodies used in this study are listed in Table S1. At least 15 mice per group were used based on power calculations for a 90% likelihood at *p* < 0.05 to see a 7.5% difference in ejection fraction with a 4.8% standard deviation for interanimal variability. Power calculations for ex vivo experiments were based on glucose content assessed in cardiomyocytes, and at least 5 samples were assayed for a 90% likelihood at *p* < 0.05 to observe a 30% difference between groups with 14% intersample variation.

### 2.2 Metabolomics

Metabolites were quantified in citrated plasma and ventricular tissue of unfasted mice using ultrahigh‐pressure liquid chromatography and triple quadrupole mass spectrometry (UPLC‐TQMS) and gas chromatography and time‐of‐flight mass spectrometry (GC‐TOFMS) as previously described, with modifications (Garcia‐Canaveras et al., 2012; Xie et al., 2013, 2015; Zhao et al., 2017). Data was processed using the TargetLynx application manager (Waters Corp., Milford, MA), and raw data from GC‐TOFMS analysis were exported in NetCDF format to ChromaTOF software (v4.50, Leco Co., CA, USA) as previously described (Garcia‐Canaveras et al., 2012; Xie et al., 2013, 2015; Zhao et al., 2017).

### 2.3 Western blotting

Protein extraction from frozen pulverized tissue of the left ventricle and isolated cardiomyocytes and western blotting and analyses were performed as previously described (Williams et al., 2018). Briefly, equal amounts of protein (20 or 30 μg) were loaded on 10% SDS‐PAGE (Tris–HCl) gels under reducing or nonreducing conditions and transferred to PVDF membranes. Intercept (PBS/TBS) Blocking Buffers (LI‐COR, cat.# 927–70,001/927–60,001) and Intercept T20 (PBS/TBS) Antibody Diluents (LI‐COR, cat.# 927–75,001/927–65,001) were used during blotting. Images were compiled for publication using Sciugo (Librach, 2023).

### 2.4 Echocardiography

We used a 38 mHz transducer with a Vevo 2100 system (Fujifilm VisualSonics) to assess the left ventricular function of sentient mice with transthoracic echocardiography. M‐mode and B‐mode images were obtained in the left ventricular parasternal short‐axis view at the level of the papillary muscle. At least three consecutive heartbeats in M‐mode were used to measure dimensions for fractional shortening (FS %) and 2D scans to obtain areas for ejection fraction (EF%). Measurements were taken blinded to the genotype and treatment.

### 2.5 RNA isolation and semiquantitative PCR

Total RNA was extracted from left ventricles using the Qiagen RNeasy kit according to the manufacturer's instructions. 1 μg of RNA was reverse transcribed using the qScript cDNA synthesis kit (QuantaBio, cat.# 95,047–100). Semiquantitative PCR (qPCR) was performed with a QuantiTect SYBR Green PCR Kit (Qiagen, cat.# 204,145) and run on a QuantStudio 12 K Flex Real‐Time PCR System (Applied Biosystems). Primers were designed to span exon‐exon junctions when possible, and sequences are provided in Table S2. Tangerin (*Ehbp1l1*) was used for normalization as its expression is unaffected by hypoxia, and its abundance is similar to our targets (Bekeredjian et al., 2010). The relative abundance of transcripts was determined by ΔΔC_t_ calculations according to standard methods.

### 2.6 RNA sequencing and analysis

Samples from male mice were used to minimize sex‐based transcriptomic differences (Yusifov et al., 2021). RNA was prepared as previously described (Williams et al., 2018). RNA samples with an RNA integrity number value ≥8 were depleted of rRNA using the NEBNext rRNA Depletion Kit v2 (New England Biolabs, cat.# E7400L). cDNA libraries were prepared according to the manufacturer's protocol (NEBNext Ultra II RNA Library Prep Kit for Illumina, New England Biolabs, cat.# E7770S). Libraries were pooled, and paired‐end sequenced on an Illumina NextSeq 550 system. Reads were quality‐checked using FastQC and prepared for alignment using PRINSEQ as previously described (Williams et al., 2018; de Sena Brandine et al., 2021; Schmieder and Edwards, 2011). Trimmed sequences were first aligned to the GRCm39 reference genome using Kallisto to determine splice variant expression of Pkm1 and Pkm2 (Bray et al., 2016). HISAT2 was used to index the *Mus musculus* UCSC mm10 reference genome and align high‐quality reads (Kim et al., 2015). The resulting sequence alignment map (SAM) files were used to count reads mapped to mouse gene models with featureCounts (Liao et al., 2013). Differential gene expression was then determined using the DESeq2 Bioconductor package (Love et al., 2014). Transcripts with a Benjamini and Hochberg *q* value <0.05, FDR <0.05, and log_2_(fold change) ≤ −1 or ≥1 (i.e., fold change ≥2) were considered differentially expressed. Differentially expressed genes (DEGs) were analyzed as previously described (Williams et al., 2018). All primary RNA‐seq data are available on Gene Expression Omnibus under accession number GSE243668. DEGs were validated using RT‐qPCR of samples from similar numbers of male and female mice.

### 2.7 Cardiomyocyte isolation

The cardiomyocyte fraction (CM) of adult mouse hearts was isolated using a Langendorff‐free method as previously described (Ackers‐Johnson et al., 2016). Buffers and media were prepared as detailed in Table S3. Briefly, high EDTA buffer was perfused through the right ventricle to inhibit contraction and coagulation and to destabilize intercellular connections. A perfusion buffer was used to remove EDTA before tissue digestion using a collagenase solution. Hearts were digested until soft and pliable for physical dissociation. Perfusion buffer containing 5% FBS was added to prevent further digestion. Cardiomyocytes were purified by sedimentation, and nonmyocytes (non‐CM) were retrieved from the supernatant. Cells were gradually reintroduced to calcium.

### 2.8 Cell viability assay

CMs were incubated in either normoxic (21% O_2_) or hypoxic (1% O_2_) conditions for 24 h in a hypoxia chamber (Baker‐Ruskinn) at 37°C. Cell viability was assessed using fluorescent dyes supplied in the Live and Dead Cell Assay (Abcam) according to the manufacturer's instructions. Cells were incubated at 37°C with 5% CO_2_ during imaging with the Leica THUNDER Imager Live Cell system. Hypoxic conditions were maintained using the Tokai Hit WSKM stage top incubator with the GM‐8000 dual‐gas mixer. Maximum projections shown. All microscopy experiments utilized both PKM2^fl/fl^ and PKM2^−/−^ groups in tandem.

### 2.9 Glucose, glycogen, and ATP assays

Cardiac glucose and glycogen were determined using the Glucose‐Glo Assay kit (Promega, cat.# J6021) and the Glycogen Assay Kit II (Abcam, cat.# ab169558), respectively. ATP was measured using the ATP Determination Kit (Invitrogen, cat.# A22066). Equivalent amounts of left ventricles were prepared for the assay according to the manufacturer's protocols. Values were normalized to total protein determined by BCA assay.

### 2.10 Glucose uptake assay

CMs were plated in M199 (Sigma‐Aldrich) supplemented with 10% FBS (Gibco) overnight, then serum starved in low glucose DMEM (Caisson) for 2 h. The media was then replaced with HEPES buffered Krebs Ringer Buffer (Boston Bioproducts, cat#. BSS‐260), supplemented with 100 nM human recombinant insulin (Sigma, cat#. 91077C) for insulin‐treated wells. Cells were then incubated with 20 μM of IRDye 800CW 2‐deoxyglucose (2‐DG, LI‐COR, cat.# 926–08946) for 1 h. After fixing with 4% paraformaldehyde, DNA was stained using TO‐PRO‐3 (Invitrogen, cat.# T3605) for normalization. 2‐DG and TO‐PRO‐3 were visualized on a LI‐COR Odyssey CLx Imaging System, and signals were quantified using Image Studio densitometry analysis software. At least 3 technical replicate wells were quantified per mouse.

### 2.11 U‐ ^13^C‐labeled glucose metabolism

Following overnight incubation in M199 (Sigma‐Aldrich) supplemented with 10% FBS (Gibco), CM media was replaced with DMEM lacking glucose and sodium pyruvate (Thermo Fisher) supplemented with 10% dialyzed FBS (Gibco) and 4.5 g/L U‐^13^C glucose (Cambridge Isotope Laboratories, cat.# CLM‐1396‐5). After 10 min, 2 h, or 18 h, cells were washed with 150 mM ammonium acetate and incubated at −80°C for 1 h with 80% methanol. 1 nmol norvaline was added as an internal standard, as previously described (Magadum et al., 2020). Cell pellets were resuspended in 0.05 mM NaOH and heated to 95°C for 20 min before protein concentration was determined. The supernatant containing the metabolites was concentrated and desiccated using a Savant SpeedVac DNA 120. Samples were then sent to the Metabolomics Core at the University of California, Los Angeles, for liquid chromatography/mass spectrometry (LC/MS).

Dried metabolites were resuspended in 50% acetonitrile (ACN) water, and 1/10th was loaded onto a Luna 3um NH2 100A (150 × 2.0 mm) column (Phenomenex) according to previously described methods. (Li et al., 2021) The chromatographic separation was performed on a Vanquish Flex (Thermo Scientific), and metabolites were detected using a Thermo Scientific Q Exactive mass spectrometer. Maven (v 8.1.27.11) was used to quantify the targeted metabolites by AreaTop using expected retention time, verified with standards, and accurate mass measurements (<5 ppm mass error). Values were normalized to the protein content of the extracted material. Relative amounts of metabolites were calculated by summing up the values for all isotopologues of a given metabolite. Metabolite Isotopologue Distributions and Fractional Contribution were corrected for natural ^13^C abundance. Data analysis was performed using in‐house R scripts, including principal component analysis and heat map generation.

### 2.12 Transmission electron microscopy and quantification of lipid droplets

Left ventricles were sectioned into 1 mm pieces at the level of the papillary muscle while submerged in fixative and fixed overnight in 2% paraformaldehyde +2.5% glutaraldehyde +2 mM CaCl_2_ in 0.1 M sodium cacodylate buffer, pH 7.4. Tissues were washed with 0.1 M cacodylate buffer and postfixed with 1% osmium tetroxide in 0.1 M cacodylate buffer for 1 h. Tissues were then dehydrated with progressively higher concentrations of ethanol before propylene oxide incubation. Resin mix (50% LX‐112, 22.2% DDSA, and 27.8% NMA) was applied before overnight incubation in a 1:1 mixture of resin mix: propylene oxide. Sections were imaged on a Hitachi HT7700 Transmission Electron Microscope. Using ImageJ, two adjacent left ventricle sections per mouse (10 images per section) were used to quantify lipid droplets normalized to total area.

### 2.13 Mitochondrial respiration and glycolysis assays

The oxygen consumption rate (OCR) and extracellular acidification rate (ECAR) of isolated CM were measured using the Seahorse XFe96 Extracellular Flux Analyzer (Agilent). CM cell viability was determined using acridine orange/propidium iodide dye (ViaStain, cat#. CS2‐0106) and analyzed using the CellDrop cell counter (DeNovix). Only live cells (8 × 10^4^ cells/well) were plated in at least 6 replicates in Seahorse media (unbuffered DMEM supplemented with 10 mM glucose, 2 mM glutamine, and 1 mM sodium pyruvate) for 1 h at 37°C in a room air incubator. HepG2 cells were used as controls to confirm treatment response, and 4 corner wells were used as temperature controls. Using the Mito Stress Test kit (Agilent, cat.# 103,015–100), oligomycin (2 μM), the mitochondrial uncoupler carbonyl cyanide‐4‐(trifluoromethoxy)phenyl‐hydrazone (FCCP, 1 μM), and rotenone/antimycin A (0.5 μM) were added after approximately 20, 40, and 60 min, respectively.

### 2.14 ROS assay

Following cardiomyocyte isolation and plating, cells were incubated in either normoxic (21% O_2_) or hypoxic (1% O_2_) conditions for 24 h. ROS levels were determined from cell pellets using the OxiSelect™ In Vitro ROS/RNS Assay Kit (Cell BioLabs, cat.# STA‐347) according to the manufacturer's protocol with H_2_O_2_ and 2′, 7′‐dichlorodihydrofluorescein (DCF) standards and normalization to total protein.

### 2.15 MitoSOX assay

CMs were incubated in either normoxic (21% O_2_) or hypoxic (1% O_2_) conditions for 24 h. Mitochondrial superoxide were stained using MitoSOX green (Thermo Fisher) according to the manufacturer's protocol. Normoxic or hypoxic conditions were maintained at 37°C using the Tokai Hit WSKM stage Top incubator described above in the cell viability assay. (Kim et al., 2011).

### 2.16 MitoTracker assay

CMs were stained with MitoTracker Red CMXRos (Thermo Fisher, cat.# M46752) according to the manufacturer's protocol. Cells were imaged as described above.

### 2.17 CaMKII activity assay

Following cardiomyocyte isolation, cell pellets were lysed using the Kinase Assay Buffer from the ADPsensor™ Universal Kinase Activity Assay Kit (BioVision, cat#. K212‐100), supplemented with Pierce™ protease inhibitor (Thermo Fisher). Small metabolites were removed from the cell lysate using a 10 kDa centrifugal filter (Amicon). Equivalent amounts of protein were used for the kinase assay, performed according to the manufacturer's instructions, using 100 μM CaMKII‐specific substrate Syntide‐2 (Cayman Chemical, cat.# 15,934) in sample reactions. Background reactions lacked ATP and Syntide‐2. Reactions were incubated at 30°C, and fluorescence was measured over time.

### 2.18 Statistical analysis

Data was analyzed using GraphPad Prism 8. Unpaired student's *t*‐test was used to compare two groups, and two‐way ANOVA with Tukey's test was used to compare more than two groups. A *p*‐value of less than 0.05 was considered significant. Error bars indicate standard deviation (SD).

## 3 RESULTS

### 3.1 Basal cardiac glucose is reduced in PKM2 ablated mice

**FIGURE 1:**
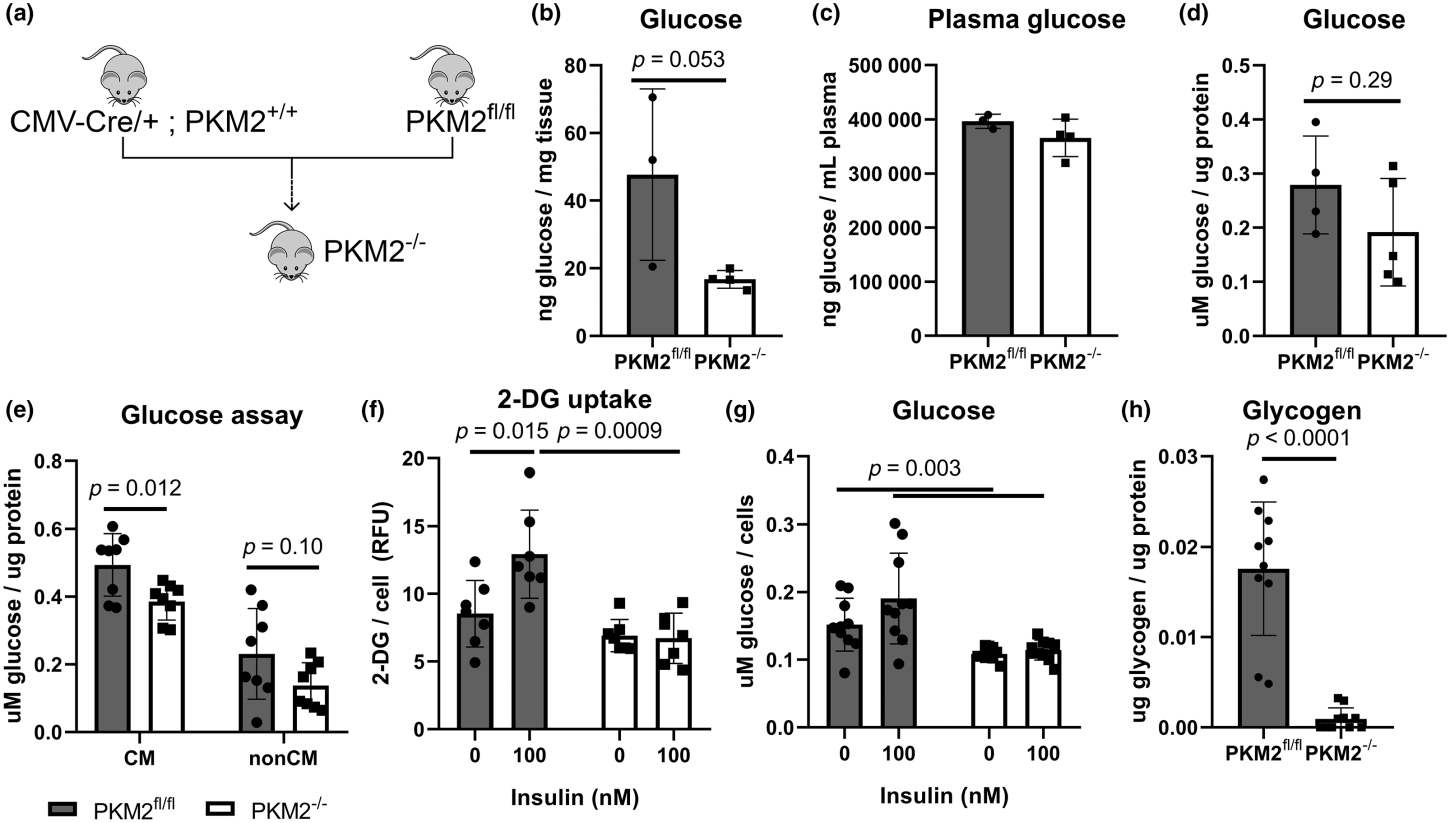
Basal and insulin‐stimulated glucose uptake is reduced in PKM2^−/−^ hearts. (a) Generation of global PKM2^−/−^ mice. (b, c) Glucose measurements in whole heart tissue and plasma (*n* = 3–4 mice per group). (d) Cardiac glucose of whole heart tissue determined by an independent glucose assay (*n* = 4–5 mice). (e) Intracellular glucose in primary CM and non‐CM (*n* = 8 mice per genotype). (f) Quantifying 2‐DG uptake in CM normalized to DNA (*n* = 7 mice per genotype). Each point represents the average of at least 3 technical replicates from a single mouse. (g) Intracellular glucose in primary CM stimulated with insulin (*n* = 10 mice per genotype). Two‐way ANOVA with Tukey's multiple comparisons. (h) Glycogen content in whole heart tissue (*n* = 10 mice per group). Student's *t*‐test versus PKM2^fl/fl^ mice unless specified. Data are shown as means ± SD.

To examine the role of PKM2 in the uninjured adult heart, we generated global PKM2 knockout (PKM2^−/−^) mice by crossing PKM2^fl/fl^ mice with mice expressing Cre driven by a CMV promoter. After germline deletion, knockout mice were bred to remove the Cre allele (Figures 1a, S1A,B). As expected, the loss of PKM2 led to a compensatory increase in *Pkm1* transcripts and PKM1 protein (Figure S1B–D).

To characterize the consequences of PKM2 loss on basal cardiac metabolism, we performed metabolomics screening on whole heart tissue and plasma from PKM2^fl/fl^ and PKM2^−/−^ mice by mass spectrometry. Given the importance of PKM2 activity in glycolysis, we were intrigued to find substantially reduced glucose in PKM2^−/−^ mouse hearts (Figure 1b). Plasma glucose levels were similar in unfasted PKM2^fl/fl^ and PKM2^−/−^ mice (Figure 1c), suggesting that the lower level in PKM2^−/−^ hearts was likely not due to changes in circulating blood sugars. We confirmed our results using an independent glucose assay and found reduced glucose in both isolated CM and nonmyocyte populations; only the reduction in CM reached statistical significance (Figure 1d,e). Although PKM2 is often associated with lactate production (Christofk et al., 2008; Israelsen & Vander Heiden, 2015), we did not observe a difference in cardiac or plasma L‐lactic acid between PKM2^fl/fl^ and PKM2^−/−^ animals, nor was there a difference in pyruvate (Figure S2). These results suggest that glucose uptake or metabolism in the heart may be dysregulated with loss of PKM2.

### 3.2 Low basal glucose levels in PKM2 ablated CMs are not due to impaired uptake

We explored potential mechanisms that might contribute to the reduction in intracellular glucose. First, we investigated glucose uptake using fluorophore‐labeled 2‐deoxyglucose (2‐DG), a glucose analog that the cell cannot metabolize. We did not observe any changes in basal 2‐DG uptake between control PKM2^fl/fl^ and PKM2^−/−^ CM (Figure 1f), although intracellular glucose levels remained lower in PKM2^−/−^ CM (Figure 1g). Interestingly, unlike PKM2^fl/fl^ CM, insulin treatment did not stimulate increased 2‐DG uptake in PKM2^−/−^ CM (Figure 1f). This was confirmed with independent measurements of intracellular glucose levels (Figure 1g). Intracellular stores can serve as another source of glucose in the cell. We quantified glycogen content in whole heart tissue and found markedly less glycogen in PKM2^−/−^ hearts than PKM2^fl/fl^ hearts (Figure 1h).

**FIGURE 2:**
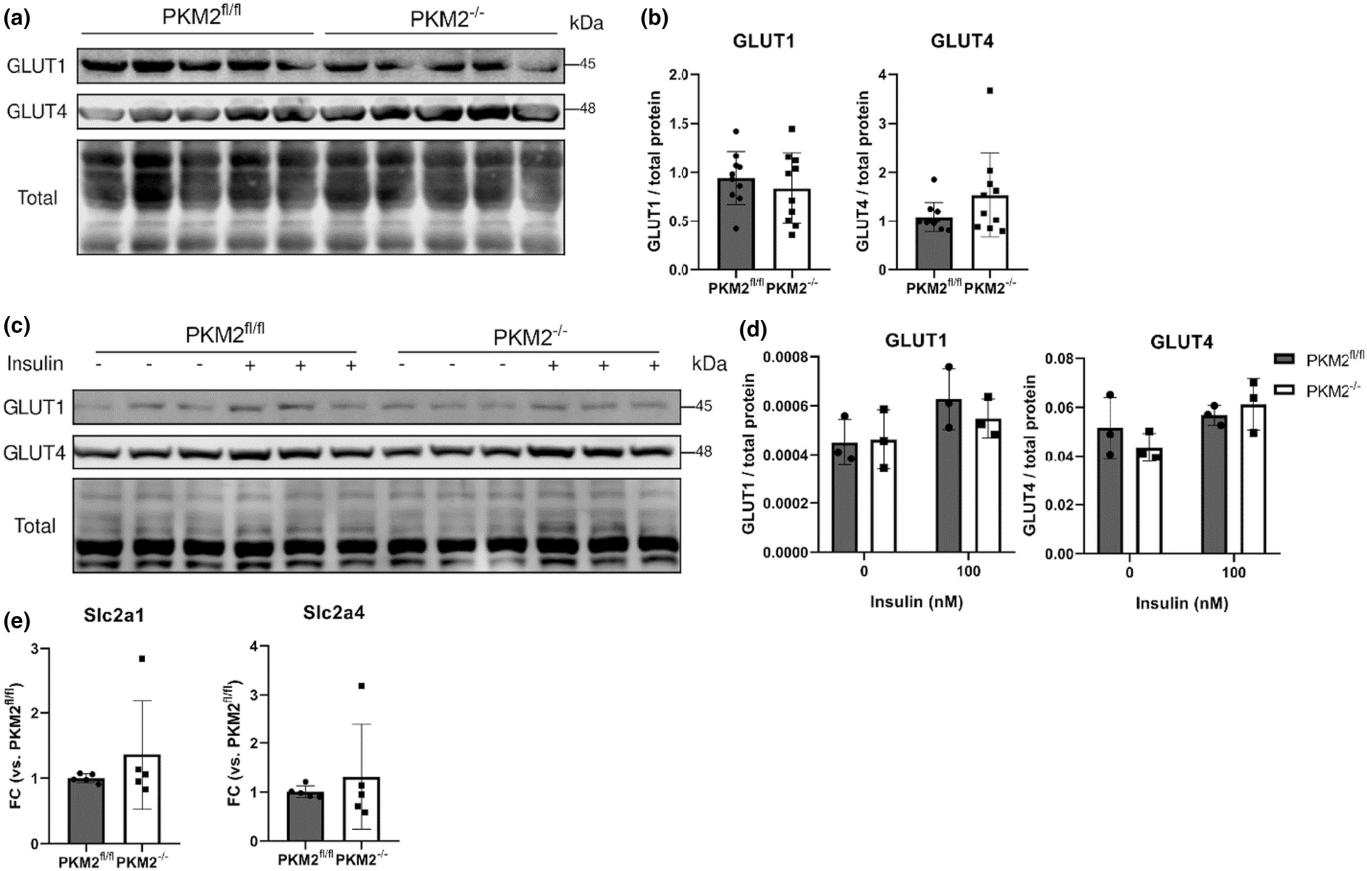
Primary cardiac glucose transporters' GLUT1/4 expressions are similar. (a, b) Western blots and quantifications of total GLUT1/4 proteins in cardiac tissue (*n* = 10 mice per group, representative blot of 5 mice per group shown, each blot normalized to total protein and PKM2^fl/fl^ controls. GLUT1, 4, and 12 were stained on the same blot. Total protein is also shown for normalization in Figure S3). (c, d) Western blot of CM incubated with or without insulin (*n* = 3 mice per genotype, normalized to total protein). Two‐way ANOVA with Tukey's multiple comparisons. (e) Slc2a1 and 4 transcripts in cardiac tissue were assessed by qPCR (*n* = 5 mice). Student's *t*‐test versus PKM2^fl/fl^ mice unless specified. Data are shown as means ± SD.

We assessed the abundance of cardiac glucose transporter to further characterize glucose uptake. We did not observe any differences in overall expression of glucose transporters 1 and 4 (GLUT1 and GLUT4) in PKM2^fl/fl^ and PKM2^−/−^ hearts (Figure 2a,b) or isolated CM, regardless of the presence or absence of insulin (Figure 2c,d), nor did we observe any differences in their transcript abundances by qPCR (Figure 2e). We also assessed GLUT3 and 12 protein abundance as these alternative hexose transporters have also been reported to be expressed in the heart, albeit at lower levels than GLUT1 and 4 (Shao & Tian, 2015). Unexpectedly, we discovered that GLUT3 was reduced in PKM2^−/−^ hearts compared to PKM2^fl/fl^ hearts (Figure S3). Despite this, similar basal 2‐DG uptake between PKM2^fl/fl^ and PKM2^−/−^ CM suggests that lower GLUT3 abundance had minimal influence on glucose uptake. Together, these data suggest that lower basal intracellular glucose in PKM2^−/−^ CM was not a result of reduced glucose delivery but instead due to increased consumption.

In addition to its direct role in glucose metabolism, PKM2 has been reported to have transcriptional activities as a nuclear coactivator of HIF‐1α and β‐catenin in various cell types, including CM, to promote the transcription of genes involved in glycolysis and proliferation (Israelsen & Vander Heiden, 2015; Magadum et al., 2020). We therefore evaluated the transcriptome of PKM2^fl/fl^ and PKM2^−/−^ hearts using RNA sequencing. Only 11 genes were differentially expressed to a degree that reached statistical significance (log_2_FC ≥1, FDR <0.05, Table S4). None were closely associated with glucose regulation, suggesting that the basal metabolic changes we observed in PKM2^−/−^ hearts are not directly due to loss of PKM2 transcriptional activity.

### 3.3 Loss of PKM2 diminishes entry of glucose‐derived metabolites into glycolysis and the TCA cycle in CM

**FIGURE 3:**
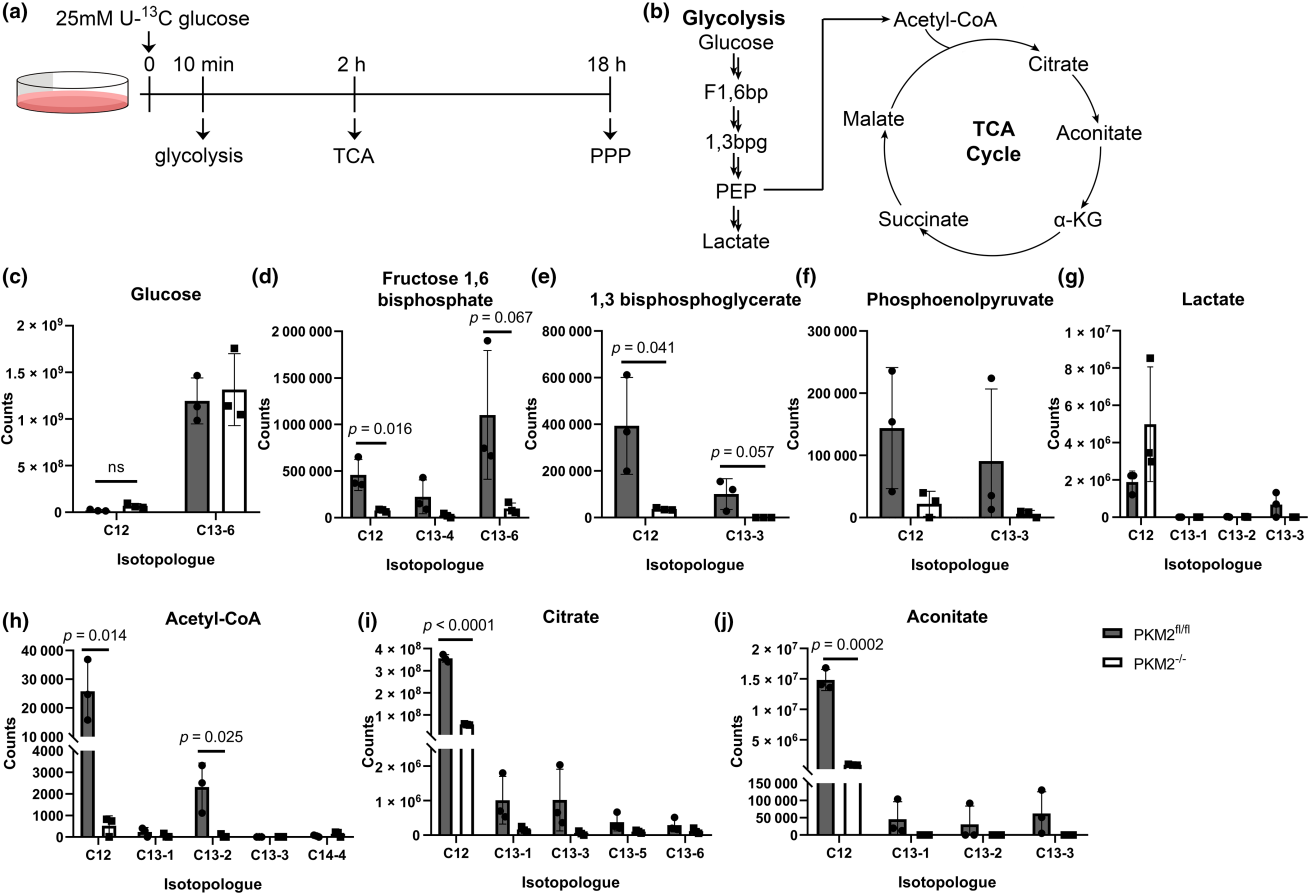
Catabolic pathways glycolysis and the TCA cycle are dysregulated in PKM2^−/−^ CM. (a) Experimental workflow. Cells are harvested 10 min, 2 h, and 18 h after the addition of U‐^13^C glucose media. (b) The schematic of glucose entry into glycolysis leads to acetyl‐CoA synthesis and the TCA cycle. (c–g) Glycolytic intermediates were assessed at 10 min of incubation. Unlabeled metabolite abundances are indicated as “C12,” labeled metabolites as “C13,” followed by the number of heavy carbons in the molecule. (h–j) TCA cycle metabolites were assessed at 2 h of incubation. All *n* = 3 mice per group. Data are shown as means ± SD. Student's *t‐*test versus PKM2^fl/fl^ mice.

We next evaluated glucose consumption as the cause of glucose depletion in PKM2^−/−^ CM. We traced the metabolism of glucose‐derived metabolites in PKM2^fl/fl^ and PKM2^−/−^ CM using uniformly labeled glucose (U‐^13^C) (Figure 3a). Specific timepoints after U‐^13^C glucose incubation were chosen based on the time required to achieve steady‐state labeling for each pathway (Magadum et al., 2020), a point at which the relative abundance of isotopically labeled metabolites remains constant over time. At 10 min post‐U‐^13^C glucose incubation, we analyzed the labeling pattern of glycolytic intermediates. TCA cycle intermediates were measured after 2 h, while PPP products were quantified after 18 h.

Similar levels of intracellular U‐^13^C glucose were observed in PKM2^fl/fl^ and PKM2^−/−^ CM (Figure 3c), suggesting basal rates of glucose uptake similar to those we saw using 2‐DG (Figure 1f). Despite similar uptake, both labeled (C13) and unlabeled (C12) glycolytic intermediates appeared to be less abundant in PKM2^−/−^ CM compared to PKM2^fl/fl^ CM (Figure 3d–g). Entry of acetyl‐CoA (Figure 3h) into the TCA cycle was also diminished in PKM2^−/−^ CM, as we observed a reduction in unlabeled citrate and aconitate (Figure 3i,j). Labeled citrate and aconitate also appeared to be reduced in PKM2^−/−^ CM compared to PKM2^fl/fl^ CM, although this did not reach statistical significance. The greater abundance of unlabeled α‐ketoglutarate, succinate, and malate in PKM2^−/−^ CM compared to PKM2^fl/fl^ CM (Figure S4) suggested that activity in later steps of the TCA cycle was maintained in PKM2^−/−^ CM, possibly through increased conversion of glutamate to α‐ketoglutarate.

### 3.4 PKM2 ^−/−^ hearts have increased lipid synthesis

**FIGURE 4:**
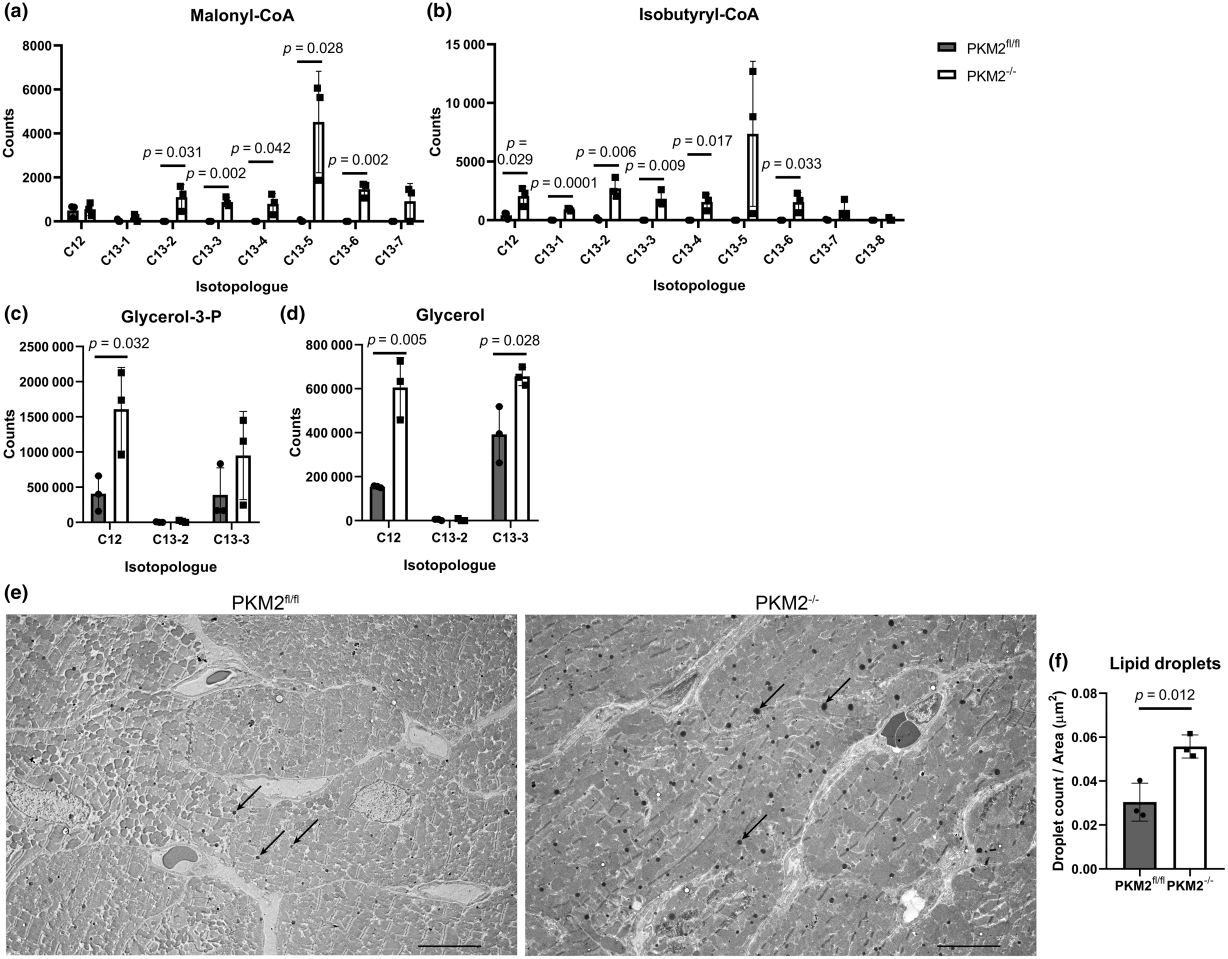
Lipid biosynthesis is dysregulated in PKM2^−/−^ hearts. (a–d) ^13^C labeling of lipid substrates in CM analyzed by LC/MS after 18 h of incubation with U‐^13^C glucose. All *n* = 3 mice per group. Data are shown as means ± SD. (e, f) Representative TEM images showing lipid droplets (black spheres, examples indicated by black arrows, *n* = 3 mice). Each dot represents the average of 10 images from one tissue section per mouse. An independent experiment of an adjacent tissue section produced similar results. Scale bar = 8 μm. Student's *t*‐test versus PKMK2^fl/fl^ mice.

Interestingly, glucose‐derived acetyl‐CoA appeared to be used for fatty acid synthesis in PKM2^−/−^ CM instead of contributing to the TCA cycle. Although labeled and unlabeled acetyl‐CoA were lower in PKM2^−/−^ compared to PKM2^fl/fl^ CM after 2 h of labeling, these metabolites were of similar abundance at 10 min of labeling, which indicated to us that the supply of acetyl‐CoA was not rate limiting (Figure S5). We observed elevated levels of U‐^13^C labeled malonyl‐CoA in PKM2^−/−^ compared to PKM2^fl/fl^ CM (Figure 4a), suggesting that glucose is incorporated into fatty acids in PKM2^−/−^ CM. Labeled isobutyryl‐CoA, which can be used in place of acetyl‐CoA to form branched‐chain fatty acids (Surger et al., 2018), was also elevated in PKM2^−/−^ CM (Figure 4b). This is in stark contrast to PKM2^fl/fl^ CMs which did not show labeling of lipids. The heart is normally not a lipogenic organ unless under metabolic stress (Bednarski et al., 2016). Similarly, we observed a greater abundance of glycerol‐3‐phosphate and glycerol (Figure 4c,d), products of dihydroxyacetone phosphate from glycolysis that can be used for triglyceride synthesis (Harding Jr. et al., 1975). We also observed almost twice as many lipid droplets in PKM2^−/−^ hearts as PKM2^fl/fl^ hearts using TEM (Figure 4e,f). Together, these data indicate that loss of PKM2 shifts cellular metabolism from oxidative metabolism to allow fatty acid synthesis in the heart.

### 3.5 PKM2 ^−/−^ CM have elevated ROS and utilize glucose in the pentose phosphate pathway

**FIGURE 5:**
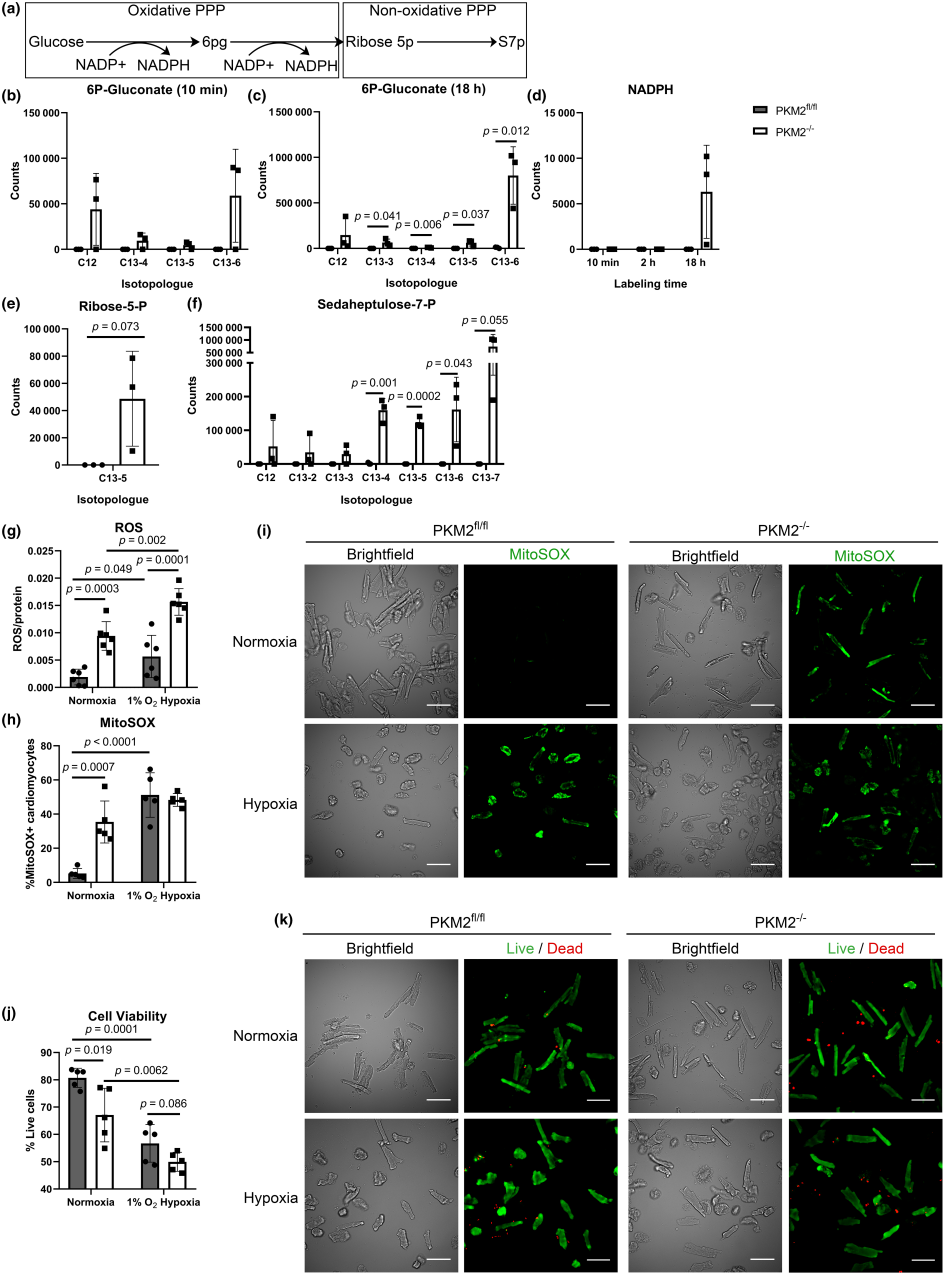
Loss of PKM2 elevates ROS and superoxide levels in CM. (a) The Schematic of glucose entry into the oxidative PPP branch generates NADPH, leading to the nonoxidative biosynthetic PPP branch. (b–f) Isotopic tracing of PPP metabolites at 18 h of incubation unless specified. 6P‐gluconate abundance at both 10 min and 18 h is shown. Total NADPH (labeled and unlabeled) was assessed across all time points. All *n* = 3 mice per group. (g) ROS (*n* = 6 mice per genotype) and (h) mitochondrial superoxide levels (*n* = 5 mice per genotype) in CM incubated at normoxia (21% O_2_) or hypoxia (1% O_2_). (i) Representative fluorescent MitoSOX images (right, green) for analysis from (h) with accompanying brightfield images on the left. Scale bar = 100 μm. (j) Quantified cell viability of isolated CM incubated at normoxia or hypoxia, (k) representative fluorescent cell viability images (right, live cells green, dead cells red) with accompanying brightfield images on the left (*n* = 5 mice per genotype). Scale bar = 100 μm. Data are shown as means ± SD. Two‐way ANOVA with Tukey's multiple comparisons.

PKM2 has been implicated in upregulating the oxidative PPP in both cardiac and cancer cells to promote the biosynthesis of lipids, proteins, and ribonucleic acids (Iqbal et al., 2013; Magadum et al., 2020). Interestingly, glucose entry to the PPP appeared virtually absent in PKM2^fl/fl^ CM. In contrast, conversion of labeled glucose to metabolites of both the oxidative (6‐phosphogluconate) and nonoxidative PPP branches (ribose‐5‐phosphate and sedaheptulose‐7‐phosphate) was abundant in PKM2^−/−^ CM (Figure 5a–f). Glucose consumption in the oxidative PPP was also observed early at 10 min of U‐^13^C glucose incubation (Figure 5b). Importantly, an increase in NADPH (labeled and unlabeled) was also observed only in PKM2^−/−^ CM after 18 h of labeling (Figure 5d), possibly from de novo synthesis and contributions from the oxidative PPP. Since NADPH can mediate ROS scavenging, (Murray, 2009) we measured ROS in isolated CM and observed higher ROS in PKM2^−/−^ CM compared to PKM2^fl/fl^ CM (Figure 5g). We also found mitochondrial superoxide was a major source of elevated ROS in the KO CM (Figure 5h,i). Increased oxidative stress was reflected in lower cell viability of isolated PKM2^−/−^ CM (Figure 5j,k). To examine the effects of environmental stress on ROS production, we exposed isolated CM to hypoxia for 24 h. We observed further increases in total and mitochondrial ROS production in both PKM2^fl/fl^ and PKM2^−/−^ CM, which correlated with further reductions in cell viability. However, the difference in mitochondrial superoxide production was lost (Figure 5g–i). These results suggest that glucose may be redirected to the PPP to mitigate the elevated ROS levels in PKM2^−/−^ CM.

### 3.6 PKM2 ^−/−^ CMs have impaired mitochondrial function and reduced ATP content

**FIGURE 6:**
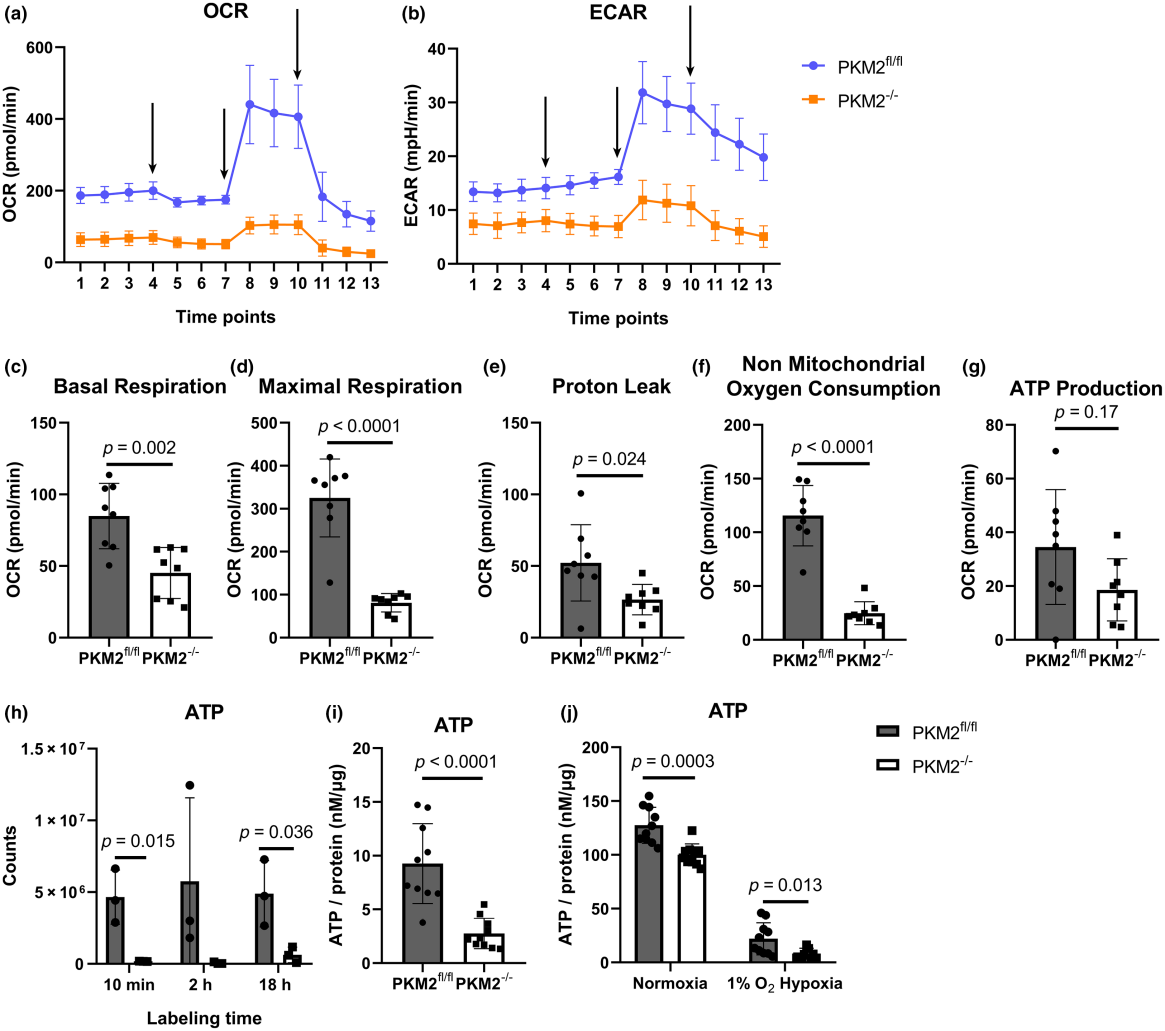
Mitochondrial respiration and ATP production are dysregulated in PKM2^−/−^ hearts. (a) OCR and (b) ECAR were measured using the Seahorse assay. Arrows indicate the addition of oligomycin, FCCP, and rotenone & antimycin A, respectively. The experiment was repeated 3 times for a total of 4 mice. Representative plot of one experiment shown; each point is the average of 8 technical replicates for one mouse. (c) Calculation of basal respiration, (d) maximal respiration, (e) proton leak, (f) nonmitochondrial oxygen consumption, and (g) ATP production as determined by Seahorse assay. Data are shown as means ± SD of 8 technical replicate wells for one mouse. Student's *t‐*test versus PKM2^fl/fl^ mice. (h) Total ATP abundance in CM determined by LC/MS across all time points (*n* = 3 mice per group). (i) ATP levels were determined in whole heart tissue (*n* = 10 mice) and (j) ATP in CM (*n* = 10 mice per genotype). Two‐way ANOVA with Tukey's multiple comparisons.

Elevated levels of mitochondrial superoxide often indicate electron leakage from the electron transport chain, resulting in inefficient oxidative metabolism (Tirichen et al., 2021). We therefore measured the oxygen consumption rate (OCR) in isolated PKM2^fl/fl^ and PKM2^−/−^ CM to evaluate mitochondrial respiration and the extracellular acidification rate (ECAR) to evaluate media acidification from glycolytic and TCA processes (lactate and CO_2_) based on glucose consumption. These parameters are reduced in the PKM2^−/−^ CM compared to PKM2^fl/fl^ (Figure 6a,b), which agrees with the isotopic labeling data of glycolytic and TCA intermediates. Additional analysis revealed proton leak, nonmitochondrial oxygen consumption, and basal and maximal respiration were all reduced in PKM2^−/−^ CM compared to PKM2^fl/fl^ CM (Figure 6c–f). Lower nonmitochondrial oxygen consumption, such as consumption by NADPH oxidase (NOX) or xanthine oxidase (XO) to generate ROS (Yung et al., 2006), may suggest that much of the ROS in PKM2^−/−^ CM at normoxia are mitochondrial, which supports the total ROS and mitochondrial superoxide results. Although PKM2 has previously been reported to regulate mitochondrial structural dynamics (Gao et al., 2022), we did not observe overt differences in mitochondrial structure in PKM2^fl/fl^ and PKM2^−/−^ CM (Figure S6A,B). We also did not observe differences in mitochondrial quantity (Figure S6C,D). Mitochondrial ATP production appears to be slightly reduced in PKM2^−/−^ CM compared to PKM2^fl/fl^ CM, as assessed by a Seahorse assay (Figure 6g), although it did not reach statistical significance. These data suggest mitochondrial dysfunction led to limited ATP production in PKM2^−/−^ CM. Quantification of total ATP levels in CM by mass spectrometry (Figure 6h, combined labeled and unlabeled) and luciferase assay in whole heart tissue and CM (Figure 6i,j) both confirmed that ATP was reduced in PKM2^−/−^ hearts and CM.

**FIGURE 7:**
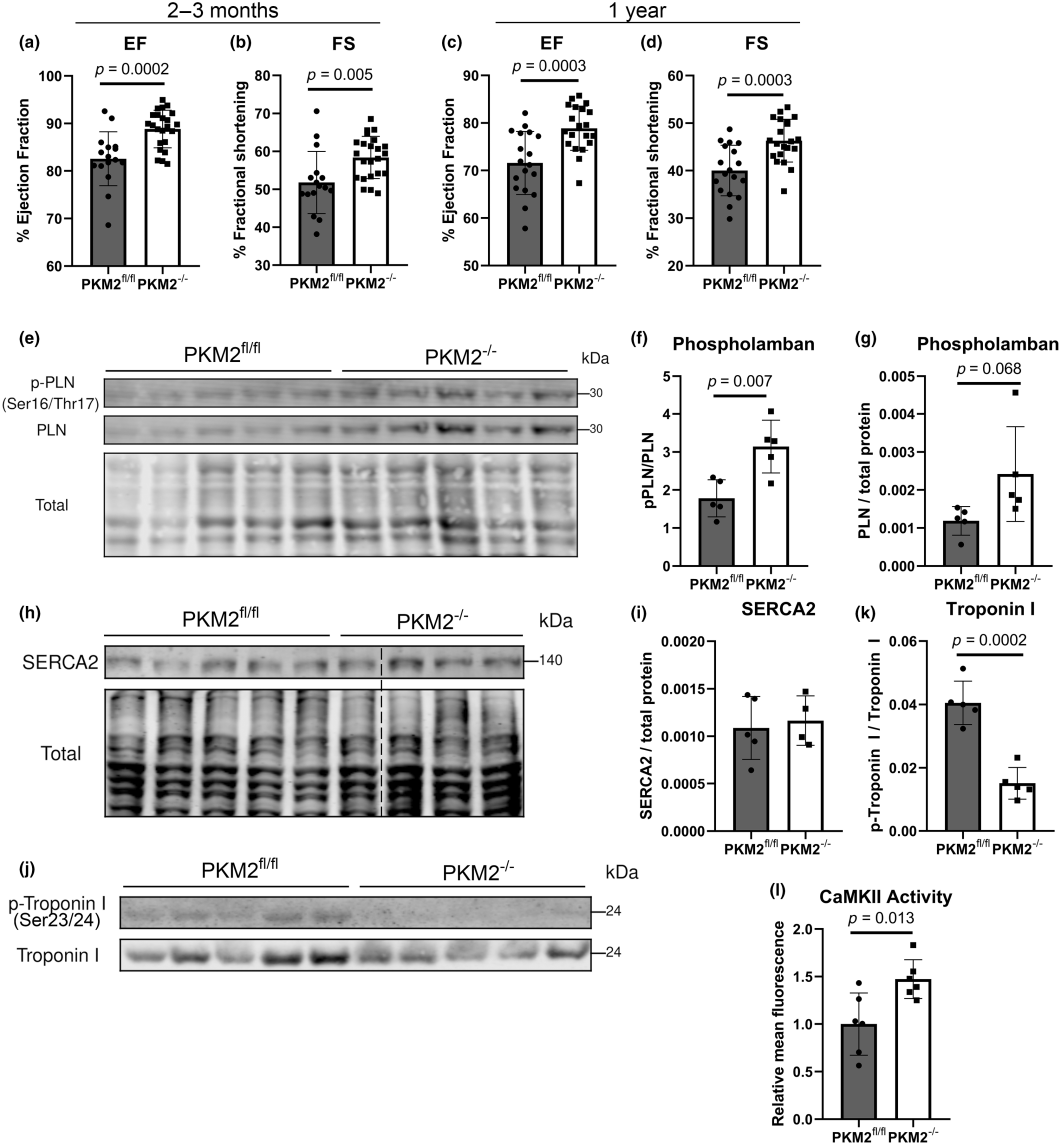
Preserved ejection fraction and fractional shortening in PKM2^−/−^ mice. (a) Ejection fraction (EF) and (b) fractional shortening (FS) assessed by echocardiography (*n* = 16 mice for PKM2^fl/fl^ and *n* = 23 for PKM2^−/−^) in mice aged 2–3 months old and (c, d) in mice aged 1 year old (*n* = 18 and 21 mice). (e–k) Western blots of phosphorylated PLN, SERCA2, and phosphorylated troponin I and their respective quantifications. Phosphorylated PLN (Ser16/Thr17) and phosphorylated troponin I (Ser23/24) normalized to PLN or troponin I, respectively (*n* = 5 mice). Total PLN (*n* = 5 mice) and SERCA2 normalized to total protein (*n* = 5 mice for PKM2^fl/fl^ and *n* = 4 for PKM2^−/−^). Dotted line in (h) represents connection of nonconsecutive lanes of the same blot. (l) CaMKII activity as assessed by kinase consumption of ATP to ADP. Data shown as means ± SD. Student's *t*‐test versus PKM2^fl/fl^ mice.

The disparity in total ATP content suggested ATP might be consumed differently in PKM2^−/−^ CM. Since the majority of ATP in cardiomyocytes is utilized for contraction and ion pumps (Doenst et al., 2013), we evaluated cardiac ejection fraction (EF) and fractional shortening (FS). Interestingly, these measurements were slightly higher in PKM2 ^−/−^ mice at 2–3 months of age (Figure 7a,b). Further assessment of cardiac geometry through measurements of the intraventricular septum (IVS), left ventricular internal diameter (LVID), and left ventricular posterior wall (LVPW) mirrored these results, with larger IVS and LVPW and smaller LVID (Figure S7). However, this did not reach statistical significance. This difference in cardiac function became more pronounced over time, with approximately a 10% greater EF in aged PKM2^−/−^ mice (>1‐year‐old, Figure 7c,d). This prompted us to investigate phospholamban (PLN) and the sarcoendoplasmic reticulum calcium ATPase 2 (SERCA2) pump, key regulators of sarcoplasmic calcium flux that influence contractility. Total PLN appeared to be increased, but importantly, we identified increased phosphorylation of PLN at Ser16/Thr17 that indicated a reduction in inhibition of the SERCA2 pump in PKM2^−/−^ hearts (Figure 7e–g) (Kranias & Hajjar, 2012). The expression of the SERCA2 pump was similar in PKM2^fl/fl^ and PKM2^−/−^ hearts (Figure 7h,i). PLN can be phosphorylated by protein kinase A (PKA) at Ser16 and calcium/calmodulin‐stimulated protein kinase II (CaMKII) at Thr17 (Kranias & Hajjar, 2012). Evaluation of another PKA target, cardiac troponin I (Ser 23/24), indicated that PKA activity was reduced in PKM2^−/−^ hearts (Figure 7j,k). Total and phosphorylated CaMKII appeared to be similar in PKM2^fl/fl^ and PKM2^−/−^ hearts (Figure S8). However, CaMKII activity was measurably higher in PKM2^−/−^ CM (Figure 7l). These results suggest that elevated CaMKII activity in PKM2^−/−^ hearts may be involved in preserving cardiac contractility by inhibitory phosphorylation of PLN. Our data showed substantial oxidative and metabolic stress in PKM2^−/−^ hearts with contractility preserved by a CAMKII‐dependent mechanism.

## 4 DISCUSSION

Metabolic homeostasis requires the coordination of energy uptake, storage, production, and expenditure. Numerous points of regulation ensure an adequate energy supply for proper cellular functions and serve as switches for adaptable metabolism in different environments. The heart's omnivorous nature and adaptable metabolism are crucial to maintaining sufficient energy production for contractility and blood circulation. Any perturbation to this system can impair heart function and exacerbate stress‐induced pathology.

Our studies revealed that loss of cardiac PKM2 leads to decreased glucose oxidation and ATP production and increased ROS generation through mitochondrial‐derived superoxide. This setting of impaired mitochondrial respiration has also been observed in several diabetic models (*db*/*db*, *ob*/*ob*, high‐fat diet). It has been linked to increased NADH and FADH_2_ flux, increased fatty acid oxidation, and subsequent mitochondrial uncoupling (Boudina et al., 2005; Teshima et al., 2014; Wright et al., 2009). High intracellular glucose can alter electron transfer donor usage to trigger mitochondrial inner membrane hyperpolarization and superoxide production. Data from early stage (Kuehne et al., 2015), diabetic mice indicate that impaired glucose utilization can precede changes in fatty acid utilization and transcription (Teshima et al., 2014).

It is unclear what the initial trigger for ROS generation may be in these mice. It is possible that in the initial healthy state of the PKM2‐ablated heart, the compensatory increase in PKM1 expression promotes oxidative metabolism to the point of excessive superoxide production, consequently altering glucose consumption in the PPP to scavenge the ROS. This would suggest that the low level of PKM2 at baseline in the heart acts as a buffer for uncontrolled oxidative metabolism. ROS may be a key factor in regulating glucose metabolism. Indeed, one study has demonstrated ROS to be a strong activator of the PPP by stimulating ATM (ataxia telangiectasia mutated) that activates glucose‐6‐phosphate dehydrogenase, redirecting glycolytic metabolites to the oxidative phase that generates NADPH, an essential cofactor for mitigating ROS (Cosentino et al., 2011; Kuehne et al., 2015). This led to reduced glucose utilization in both glycolysis and the TCA cycle. We also observed a reduction of early TCA cycle metabolites and an increase of unlabeled TCA metabolites in PKM2^−/−^ cardiomyocytes beginning at α‐ketoglutarate, which implies the utilization of an unlabeled source. Glutamine and glutamate can enter the TCA cycle once converted to α‐ketoglutarate, as can other amino acids at various points. The increase in unlabeled α‐ketoglutarate, succinate, and malate and the absence of labeling on α‐ketoglutarate and succinate further suggests that glucose is not being utilized in oxidative metabolism. Our data suggest that PKM2 regulates basal cardiac metabolism and oxidative stress. It will be important in future studies to identify the cause of ROS production in PKM2^−/−^ hearts in anticipation of potential therapeutic interventions.

We also present evidence of preserved cardiac contractility in global PKM2^−/−^ mice. Inhibitory phosphorylation of PLN was increased in PKM2^−/−^ hearts, possibly by CaMKII, allowing for enhanced SERCA2 activity, which may increase ATP consumption. CaMKII can be activated by oxidation of residues Cys281/Met282 or Met281/Met282, as demonstrated by induction with hydrogen peroxide (Erickson et al., 2008; Howe et al., 2004; Rocco‐Machado et al., 2022; Sanders et al., 2013). The concurrent increase in ROS and CaMKII activity in PKM2^−/−^ hearts may indicate CaMKII oxidation, suggesting that the preserved contractility in PKM2^−/−^ hearts is another consequence of oxidative stress. In support of this, ROS has been previously documented to modify sarcoplasmic calcium flux, which has led to altered contractile function (Wagner et al., 2014; Zhang et al., 2015). While SERCA2 directly regulates diastolic function of the heart, other studies have shown that increasing SERCA2 abundance and activity can increase ejection fraction and fractional shortening (Federica del et al., 1999; Xin et al., 2017). This positive inotropic effect, observed with SERCA2 activation via β‐adrenergic signaling, may result from increased calcium accumulation in the sarcoplasmic reticulum, which enhances calcium release in the subsequent contraction cycle (Eisner et al., 2017). Several components are likely contributing to this effect. A more detailed assessment of the contractile machinery will be necessary to fully elucidate the mechanism of preserved contractility in PKM2^−/−^ hearts.

We also found other diabetic indicators in PKM2^−/−^ hearts, including impaired insulin‐mediated glucose uptake, increased fatty acid synthesis, and lipid accumulation. The relationship between cardiac lipid accumulation and heart failure has been well documented (Schulze et al., 2016), where resulting cardiac lipotoxicity has been associated with impaired heart function (Boudina & Abel, 2007; Szczepaniak et al., 2003), insulin resistance (Patel et al., 2016), and mitochondrial dysfunction (Boudina et al., 2012; Sparks et al., 2005). Previous studies have also shown that the accumulation of fatty acids such as malonyl‐CoA in rat hearts can inhibit fatty acid oxidation (Saddik et al., 1993). The link between mitochondrial dysfunction and increased fatty acid synthesis with reduced β‐oxidation has been demonstrated in 3 T3‐L1 cells, showing intracellular accumulation of triglycerides upon impairment of mitochondrial respiration (Vankoningsloo et al., 2005). Increased diacylglycerols and ceramides have also been observed in diabetic models and contribute to impaired insulin signaling and diabetic cardiomyopathy (Jia et al., 2018). Taken together, these studies suggest that loss of PKM2 in the heart may disrupt metabolism and energy production, with each component exacerbating the effects of the other. This appears to be tolerated in otherwise healthy mice for substantial periods but eventually accumulates to the point that cardiac function is impaired due to metabolic stress in aged mice. A previous study found that aged PKM2^−/−^ mice spontaneously develop insulin resistance and hepatic steatosis, which led to hepatocellular carcinoma (Dayton et al., 2016).

Cardiac mitochondria are considered resilient compared to those in other tissues, based on high respiratory efficiency and energy production with low peroxide presence, even in aged hearts (Brandt et al., 2017). It is thought that cardiomyocytes have mechanisms to combat oxidative stress and maintain contractility (Kurian et al., 2016) and thus may not develop pathological changes without additional stressors. Indeed, cardiomyocyte‐specific PKM2 ablation has demonstrated elevated ROS correlated with increased apoptosis, but only in the presence of doxorubicin (Saleme et al., 2019). Our results support these findings with hypoxic PKM2^−/−^ cardiomyocytes having decreased viability compared to PKM2^fl/fl^ controls (Figure 5j,k). Combined with our mitochondrial functional data, these results suggest that loss of PKM2 may limit cellular resilience to metabolic stress (as indicated by lower maximal mitochondrial respiration) and leave cardiomyocytes more susceptible to injury from environmental stress.

There is a growing emphasis on evaluating cardiometabolic risk using biomarkers that precede the onset of disease (Mietus‐Snyder et al., 2023). When a patient presents with clinical symptoms, it can be difficult to intervene in a way that limits disease progression. Cardiac energetics have emerged as potential predictors for heart failure risk, focusing on creatine kinase (CK) as it rapidly supplies ATP during high‐energy demand. Our findings suggest that PKM2 is important in maintaining energy stores in cardiomyocytes and that loss of basal PKM2 function causes metabolic stress in the heart. Due to metabolic and transcriptional activities, PKM2 has been implicated in several cardiovascular diseases as a cardioprotective enzyme (Rihan & Sharma, 2023). Cardiomyocyte‐specific PKM2 deficiency increased apoptosis of cardiomyocytes due to loss of PKM2 transcriptional activity, leading to the development of fibrosis and dilated cardiomyopathy in an age‐dependent manner in mice (Lorenzana‐Carrillo et al., 2022). Cardiac‐specific PKM2 deletion has been demonstrated to exacerbate cardiac function and remodeling under pressure overload, while PKM2 overexpression improved cardiac recovery (Ni et al., 2022). These findings suggest that PKM2 improves energy utilization in a manner that can ameliorate cardiac pathology.

A limitation of our study is that PKM2 is ablated in all cells in our mice. This may present confounding effects when studying cardiac‐specific events. Further study using cell‐specific deletions of PKM2 will be important to delineate the specific contribution of PKM2 in each cell type within the heart and its effect on function. However, global knockout of PKM2 can also serve as a model for systemic metabolic and oxidative stress that closely resembles nonalcoholic fatty liver disease, where symptoms in mice were preceded by metabolic dysfunction in the liver (Dayton et al., 2016). Additionally, conditions that may inhibit PKM2 function or drugs that alter PKM2 activity would have systemic effects as PKM2 is ubiquitously expressed. Indeed, a PKM2 activator is currently under clinical investigation as an anti‐cancer therapy (NCT04328740). Therefore, our results may shed light on cardiac side effects from these potential treatments.

The global knockout model also makes it difficult to distinguish the effects of PKM2 deletion in other tissues that contribute to both systemic and cardiac metabolism. For example, PKM2 ablation has been demonstrated to reduce insulin secretion from β‐cells (Foster et al., 2022), which may have secondary effects on insulin signaling. Although PKM2 is typically expressed at very low levels in the heart compared to other tissues (Taniguchi et al., 2015), our data from isolated cardiomyocytes indicate that loss of PKM2 in these cells is sufficient to alter cellular metabolism. Future studies using tissue‐specific PKM2 knockout mice will be needed to determine the impact of PKM2 deletion in metabolic tissues on cardiac function and metabolism.

Another concern is that metabolic measurements in isolated cells may not accurately reflect processes that occur in vivo. However, our results from isolated CM largely agreed with data collected from whole heart tissue, indicating our observations were not likely a consequence of our in vitro measurements or PKM2 deletion in other cardiac cells. Whereas the sample sizes in our in vivo experiments were larger, some of the in vitro experiments were relatively small and therefore limit the statistical power of these results. However, these data were all corroborated through independent assays.

Our findings indicate that PKM2 regulates basal cardiac metabolism by preventing excessive ROS generation and maintaining ATP production. These results may help explain why overexpression of PKM2 can be protective after injury such as myocardial infarction and indicate that modulation of PKM2 activity may hold promise as a therapeutic intervention to limit oxidative stress and preserve ATP production in heart disease.

## AUTHOR CONTRIBUTIONS

RVS, ALW, KCYL conceived and designed research. KCYL, ALW, LW, GX, AF performed experiments, KCYL, ALW, LW, GX, WJ, AF, MG analyzed results, and KCYL, ALW, AF, MG, RVS interpreted results of experiments. KCYL drafted the manuscript and prepared figures, RVS, ALW, KCYL revised and edited the manuscript. KCYL, ALW, LW, GX, WJ, AF, MG, RVS approved the final version of the manuscript.

## FUNDING INFORMATION

This work was supported by the National Institutes of Health [grant numbers P30 GM103341 to RVS, T32 HL115505 to ALW and KCYL, P20 GM103451 to MG, P30 CA‐071789 and 5G12 MD‐007601 to the Microscopy and Imaging Core, P20 GM‐103466, U54 GM138062, and 5P30 GM‐114737 to the Bioinformatics Core, Shared Instrumentation grants 1S10 OD016387 (T.G.G.) to the UCLA Metabolomics Core and 1S10OD028515–01 to the MIFCC, and an Institutional Development Award (IDeA) P20GM103451 to the NM‐INBRE]; and the American Heart Association [grant number 23PRE1027246 to KCYL].

## CONFLICT OF INTEREST STATEMENT

The authors declare no conflicts of interest.

## ETHICS STATEMENT

All animal protocols and experiments were approved by the Institutional Animal Care and Use Committee of the University of Hawaii at Manoa (IACUC approval number 06‐011‐17).

## Supporting information


Figure S1.

Table S1.

## Data Availability

RNA‐seq data are available in the National Center for Biotechnology Information Gene Expression Omnibus Repository (series GSE243668). Data are also available from the corresponding author upon reasonable request.
