# Correction to “PKM2 regulates metabolic flux and oxidative stress in the murine heart”

**DOI:** 10.14814/phy2.70100

**Published:** 2024-10-24

Lee, K. C. Y., Williams, A. L., Wang, L., Xie, G., Jia, W., Fujimoto, A., Gerschenson, M., & Shohet, R. V. (2024). PKM2 regulates metabolic flux and oxidative stress in the murine heart. *Physiological Reports*, *12*(17), e70040.

In the “Funding Information” section, the text “P20 GM103451 to MG” was incorrect. This should have read “P20 GM113134 and U54MD007601 to MG.”

We apologize for this error.

