# Supplementary material for: PKM2 regulates metabolic flux and oxidative stress in the murine heart
Source: Physiol Rep. 2024 Sep 10;12(17):e70040. doi: 10.14814/phy2.70040 (PMC11387154; doi:10.14814/phy2.70040)
Supplement: Supplementary file 1 — Figure S1. [file PHY2-12-e70040-s002.docx]

FIGURE S1


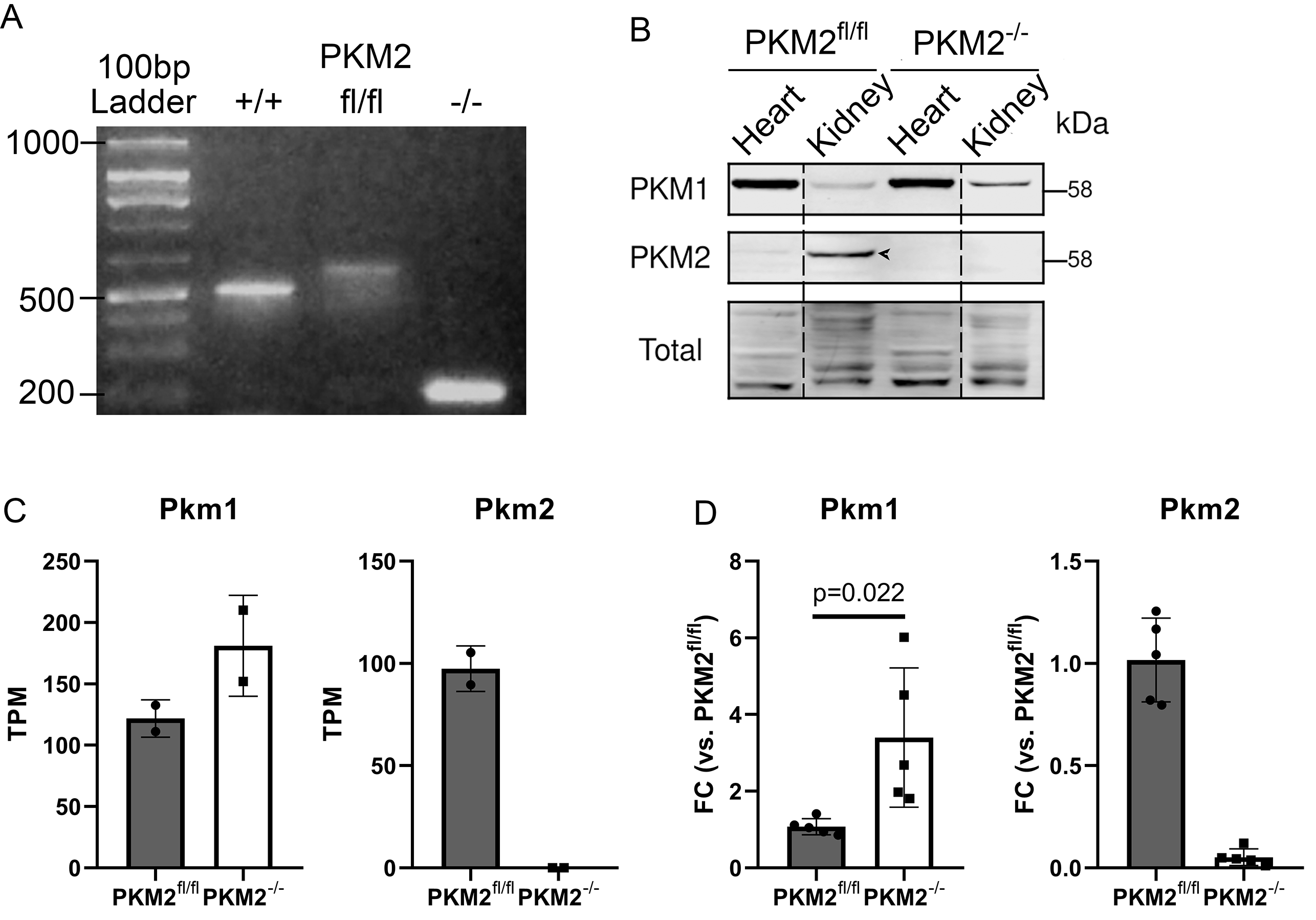


Figure S1. PKM2 ablation increases Pkm1 expression.

(*A*) Polymerase chain reaction with primers surrounding exon 10 (Pkm2) excised in PKM2^-/-^ mice. Lane 1 – ladder, lane 2 – PKM2^+/+^ (wildtype), lane 3 – PKM2^fl/fl^, lane 4 – PKM2^-/-^. (*B*) Western blot showing PKM2 ablation and PKM1 protein in global PKM2^-/-^ mice. The total protein is shown for normalization. Dotted lines represent the connection of non-consecutive lanes of the same blot. Arrowhead indicates PKM2 presence in PKM2^fl/fl^ mice and deletion in PKM2^-/-^ mice. (*C*) *Pkm1* and *Pkm2* transcript abundances determined by RNA-seq (n=2-3 mice per group), plotted by transcripts per million (TPM) and (*D*) qPCR (n=5 mice per group). Data are shown as means ± SD. Student’s *t-*test vs. PKM2^fl/fl^ mice.

FIGURE S2


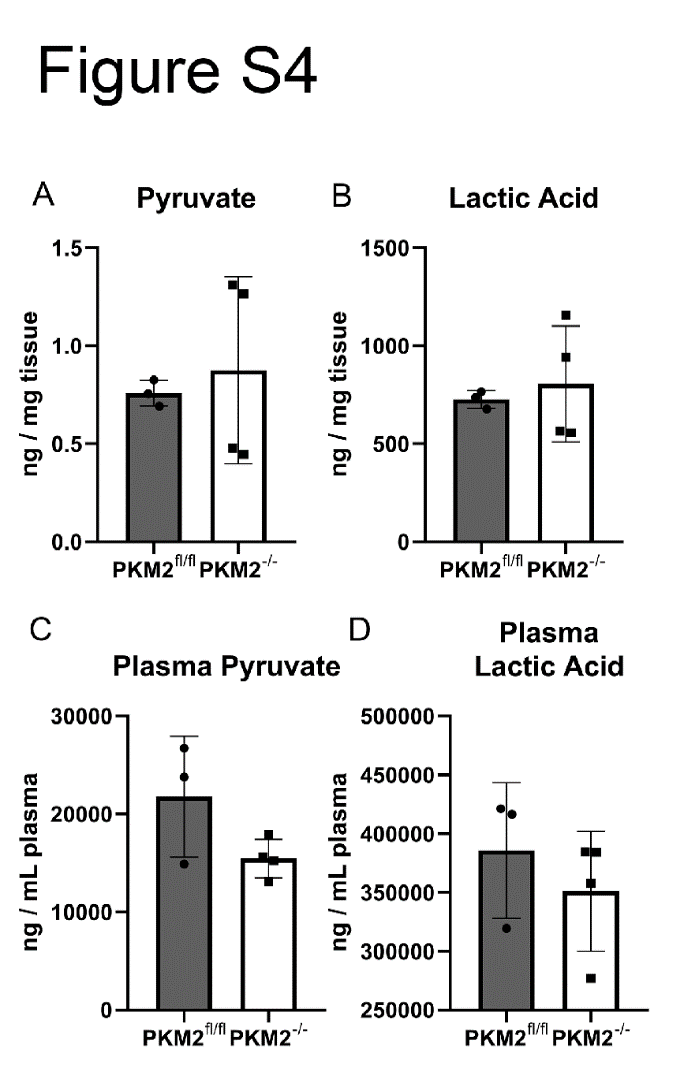


Figure S2. Pyruvate and lactate levels in PKM2^fl/fl^ and PKM2^-/-^ hearts and blood.

(*A-B*) Pyruvate and lactate measurements in whole heart tissue and (*C-D*) plasma (n=3-4 mice per group). Data are shown as means ± SD.

FIGURE S3


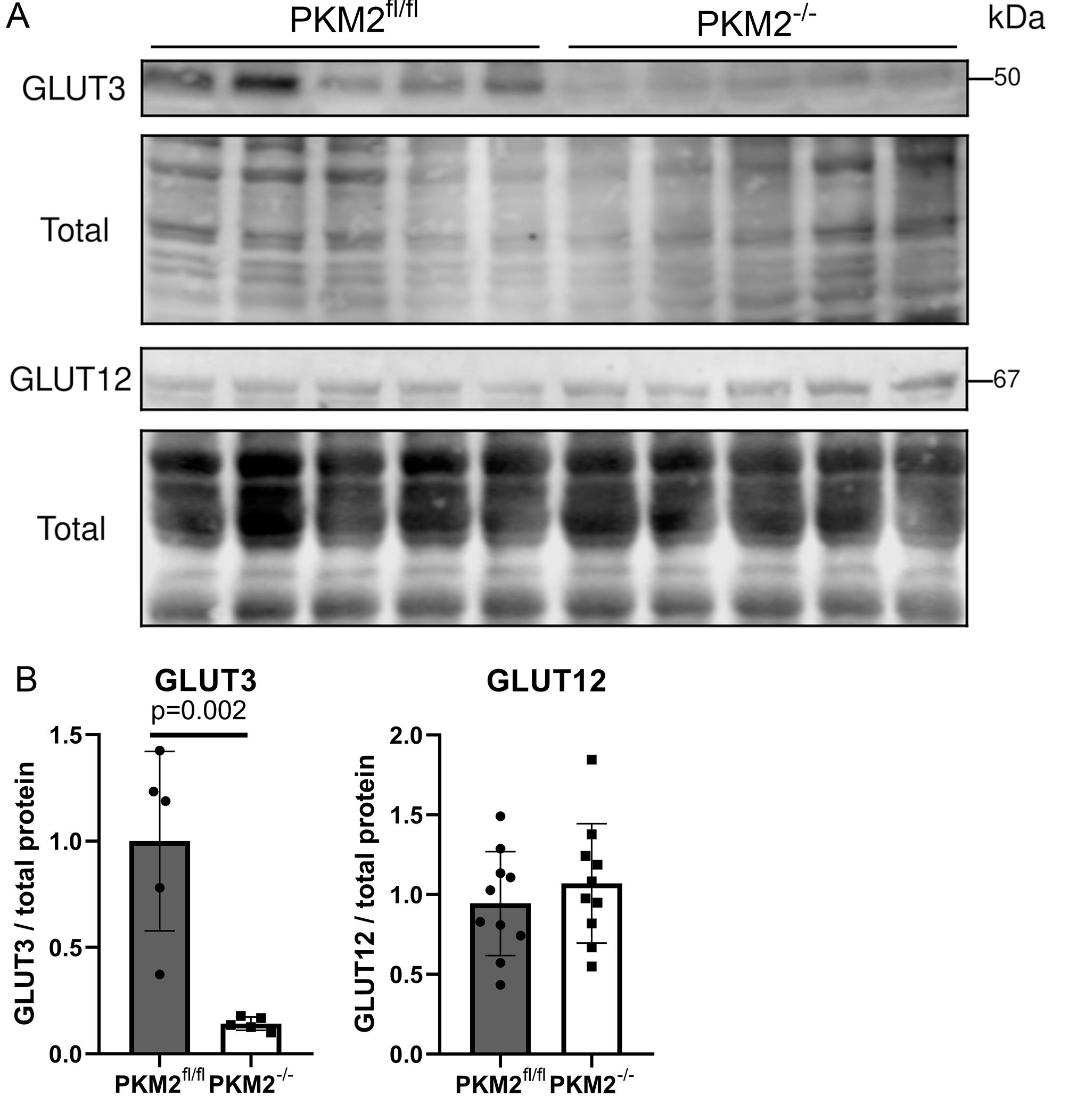


Figure S3. The protein abundance of hexose transporters is similar in PKM2^fl/fl^ and PKM2^-/-^ hearts.(*A-B*) Western blot and quantifications of GLUT3 and 12 in PKM2^fl/fl^ and PKM2^-/-^ cardiac tissue (n=5 per group. GLUT12 was repeated for n=10, and a representative blot was shown. Each blot normalized to total protein and PKM2^fl/fl^ controls. GLUT1, 4, and 12 were stained on the same blot. Total protein is also shown for normalization in Figure 2). Data are shown as means ± SD. Student’s *t-*test vs. PKM2^fl/fl^ mice.

FIGURE S4

Figure S4. Increased abundance of unlabeled later TCA cycle intermediates in PKM2^-/-^ CM.

(*A-C*) TCA cycle metabolites were assessed at 2 hours of incubation. All n=3 mice per group. Data are shown as means ± SD. Student’s *t-*test vs. PKM2^fl/fl^ mice.

FIGURE S5


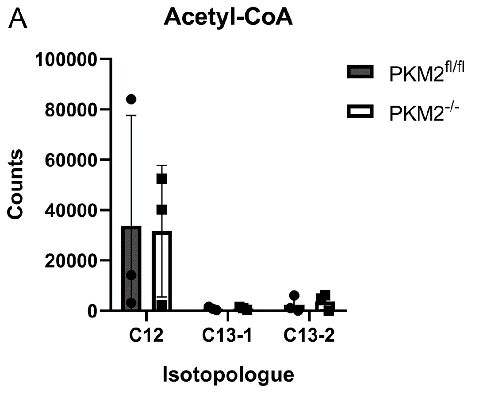


Figure S5. Production of acetyl-CoA is similar between PKM2^fl/fl^ and PKM2^-/-^ CM after 10 minutes of labeling.

(*A*). Isotopic tracing of acetyl-CoA at 10 minutes after incubation (n=3 mice per group). Data shown as means ± SD.

FIGURE S6


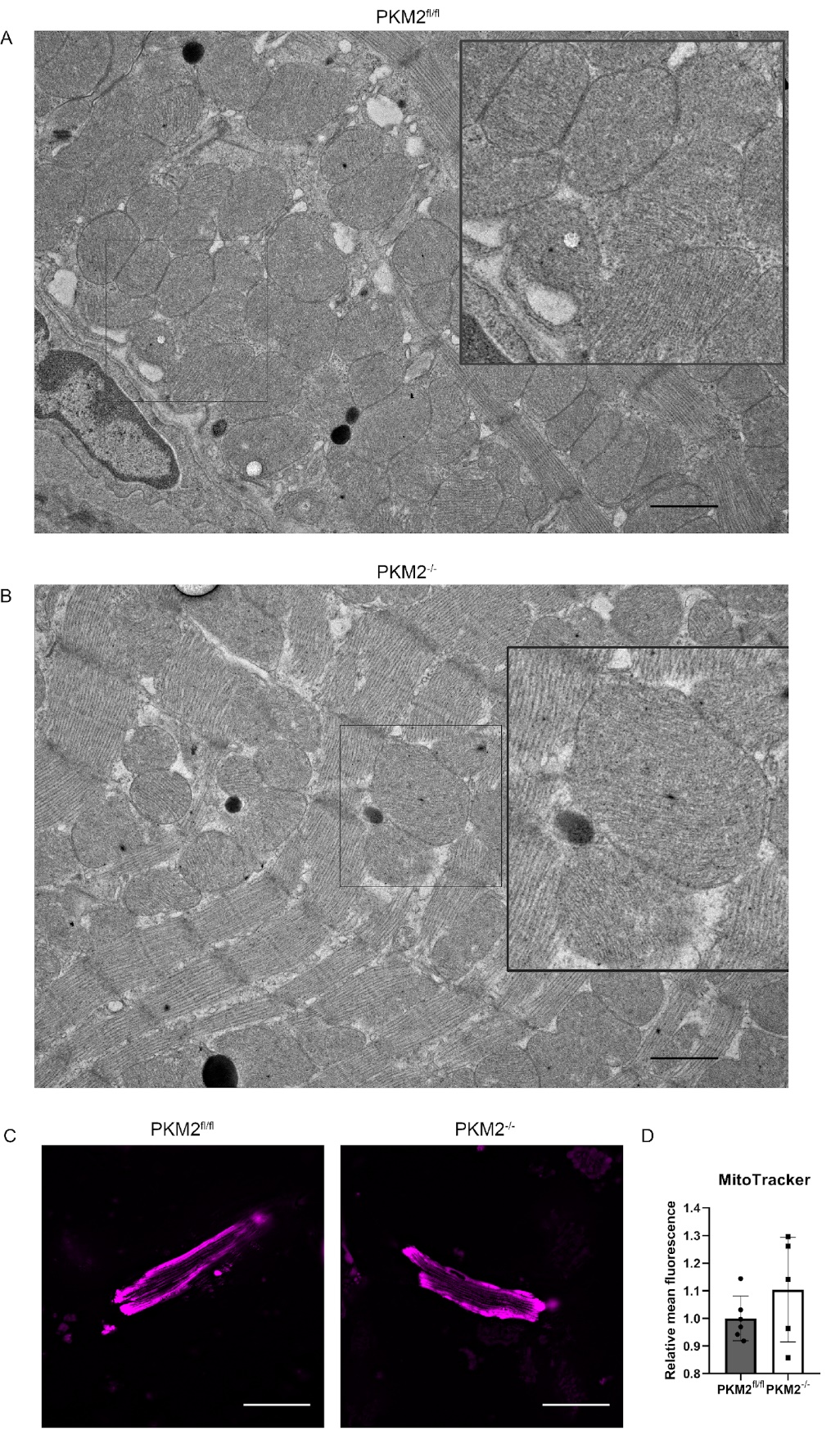


Figure S6. Mitochondrial structure is similar in PKM2^fl/fl^ and PKM2^-/-^ hearts.

(*A*) Representative TEM images of mitochondria in PKM2^fl/fl^ and (*B*) PKM2^-/-^ hearts. Insets show high magnification of the regions indicated. Scale bar = 1μm. (*C*) Representative MitoTracker images in PKM2^fl/fl^ and PKM2^-/-^ cardiomyocytes. Scale bar = 50μm. (*D*) Quantification of MitoTracker in cardiomyocytes (n=5 mice per group). At least 100 cardiomyocytes were measured per mouse. Data are shown as means ± SD.

FIGURE S7


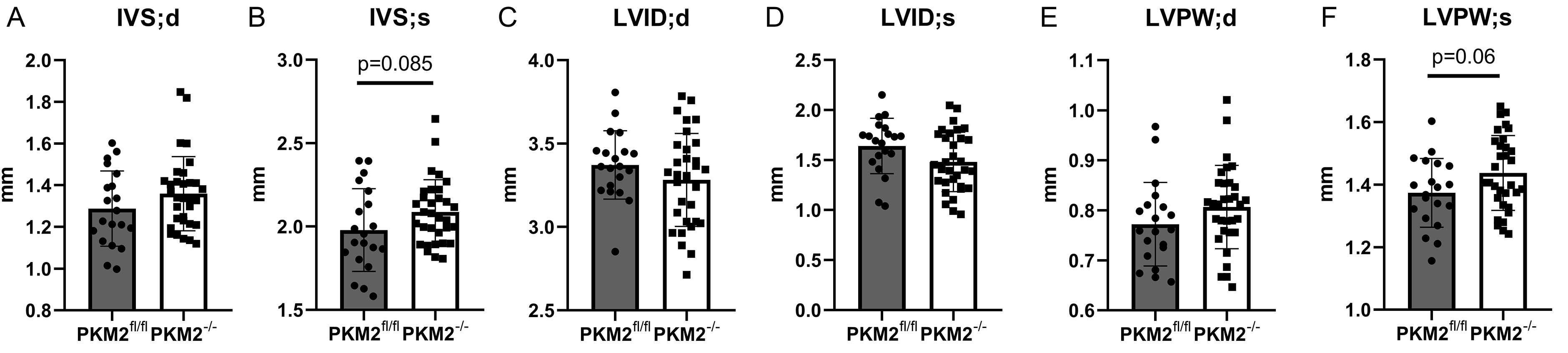


Figure S7. Additional echocardiogram measurements.

Dimensions at diastole (d) and systole (s) of (*A-B*) the intraventricular septum (IVS), (*C-D*) left ventricular internal diameter (LVID), and (*E-F*) left ventricular posterior wall (LVPW) (n=20 PKM2^fl/fl^ mice and n=32 PKM2^-/-^ mice). Data are shown as means ± SD. Student’s *t-*test vs. PKM2^fl/fl^ mice.

FIGURE S8


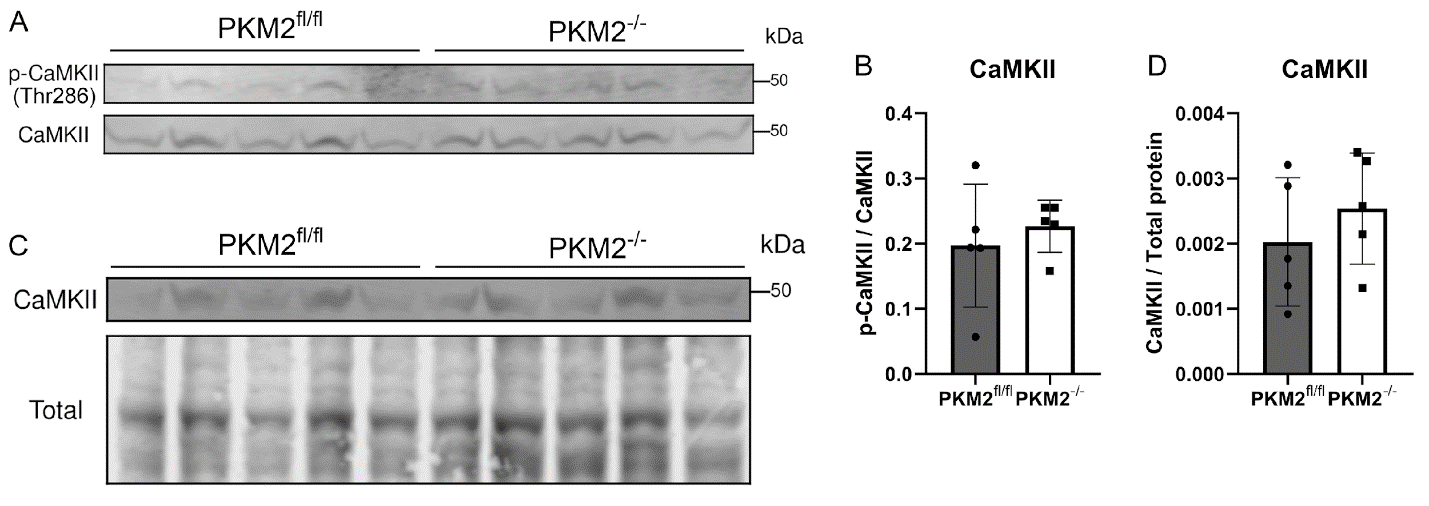


Figure S8. Total CaMKII protein and phosphorylation of whole heart lysate.

(*A-B*) Western blot and quantification of phosphorylated CaMKII (Thr286). (*C-D*) Nonreducing western blot and quantifying CaMKII, normalized to total protein (n=5 mice per group). Data are shown as means ± SD.
