# Supplementary material for: PKM2 regulates metabolic flux and oxidative stress in the murine heart
Source: Physiol Rep. 2024 Sep 10;12(17):e70040. doi: 10.14814/phy2.70040 (PMC11387154; doi:10.14814/phy2.70040)
Supplement: Supplementary file 2 — Table S1. [file PHY2-12-e70040-s001.docx]

| **Table S1: Antibodies used in this study** | | | | |
| --- | --- | --- | --- | --- |
| **Antigen** | **Dilution** | **Company** | **Catalog number** | **RRID** |
| GAPDH | WB 1:20000 | Sigma | G8795 | AB_1078991 |
| PKM1 | WB 1:4000 | Proteintech | 15821-1-AP | AB_2163820 |
| PKM2 | WB 1:4000 | Proteintech | 15822-1-AP | AB_1851537 |
| GLUT1 | WB 1:1000 | Proteintech | 21829-1-AP | AB_10837075 |
| GLUT4 | WB 1:2000 | Proteintech | 66846-1-Ig | AB_2882186 |
| GLUT3 | WB 1:1000 | Santa Cruz Biotechnology | sc-74497 | AB_1124974 |
| GLUT12 | WB 1:1000 | Thermo Fisher Scientific | PA5-80020 | AB_2747135 |
| Phospho-Troponin I (Cardiac) (Ser23/24) | WB 1:1000 | Cell Signaling | 4004 | AB_2206275 |
| Troponin I | WB 1:1000 | Cell Signaling | 4002 | AB_2206278 |
| SERCA2 | WB 1:1000 | Invitrogen | MA3-919 | AB_325502 |
| Phospho-PLN (Ser16/Thr17) | WB 1:1000 | Cell Signaling | 8496 | AB_10949102 |
| PLN | WB 1:1000 | Cell Signaling | 8495 | AB_10949105 |
| Phospho-CaMKII (Thr286) | WB 1:1000 | Cell Signaling | 3361 | AB_10015209 |
| CaMKII | WB 1:2000 | Invitrogen | MA1-048 | AB_325403 |
| IRDye 800CW Donkey anti-Rabbit IgG | WB 1:20000 | LI-COR | 926-32213 | AB_621848 |
| IRDye 680RD Donkey anti-Mouse IgG | WB 1:20000 | LI-COR | 926-68072 | AB_10953628 |

Table S1. Antibodies used in this study.

Primary and secondary antibodies used for western blotting listed with Research Resource Identifiers (RRID).

| **Table S2: Primer sequences** | | | |
| --- | --- | --- | --- |
| Gene | Forward | Reverse | Length (bp) |
| Pkm1 | CACCGTCTGCTGTTTGAAGA | AGCACTCCTGCCAGACT | 144 |
| Pkm2 | CATCTACCACTTGCAGCTATTC | GAGCACTCCTGCCAGACT | 152 |
| Glut1 | GATTGGTTCCTTCTCTGTCGG | ATCTCAAAGGACTTGCCCAG | 132 |
| Glut4 | GTAACTTCATTGTCGGCATGG | AGCTGAGATCTGGTCAAACG | 155 |
| Ehbp1l1 | TTCCAGTTTGTGGCGTGTTAC | TTCCGCCGAGTCCATACCA | 91 |
| Pkm2 | TAGGGCAGGACCAAAGGATTCCCT | CTGGCCCAGAGCCACTCACTCTTG | Note: For genotyping |

Table S2. Primer sequences.

Primers used for qPCR unless otherwise specified. qPCR primers were designed to span exon-exon junctions when possible.

| **Table S3: Cardiomyocyte isolation buffers** | |
| --- | --- |
| **EDTA buffer** | |
| **Reagent** | **Final concentration** |
| MilliQ-dH_2_O | - |
| NaCl | 130mM |
| KCl | 5mM |
| NaH_2_PO_4_·H_2_O | 0.5mM |
| HEPES | 10mM |
| Taurine | 10mM |
| EDTA | 10mM |
| D-glucose | 10mM |
|  |  |
| **Perfusion buffer** | |
| **Reagent** | **Final concentration** |
| MilliQ-dH_2_O | - |
| NaCl | 130mM |
| KCl | 5mM |
| NaH_2_PO_4_·H_2_O | 0.5mM |
| HEPES | 10mM |
| Taurine | 10mM |
| MgCl_2_ | 1mM |
| D-glucose | 10mM |
|  |  |
| **Plating Media** | |
| **Reagent** | **Final concentration** |
| M199 media | - |
| FBS | 5% |
|  |  |
| **Culture Media** | |
| **Reagent** | **Final concentration** |
| M199 media | - |
| BSA | 0.1% |
| ITS | 1% |
| CD lipid | 1% |

Table S3. Cardiomyocyte isolation buffers.

Buffer and media formulations for Langendorff-free cardiomyocyte isolation.

| **Table S4: RNA-seq DEGs** | | | | | | | | | |
| --- | --- | --- | --- | --- | --- | --- | --- | --- | --- |
| Gene name | log_2_FoldChange | lfcSE | stat | pvalue | padj | PKM2^fl/fl^ | PKM2^fl/fl^ | PKM2^-/-^ | PKM2^-/-^ |
| Gm20594 | 3.7 | 0.4 | 9.1 | 7.5E-20 | 2.0E-15 | 108 | 181 | 1964 | 1796 |
| Rny1 | 2.6 | 0.4 | 7.2 | 7.6E-13 | 1.0E-08 | 450 | 728 | 3049 | 4182 |
| Snap91 | 2.9 | 0.6 | 5.2 | 2.2E-07 | 2.0E-03 | 39 | 23 | 181 | 279 |
| Cd44 | 4.3 | 0.9 | 4.8 | 2.0E-06 | 9.9E-03 | 2178 | 4265 | 64037 | 65838 |
| Lars2 | 4.6 | 1.0 | 4.7 | 2.2E-06 | 9.9E-03 | 11991 | 24451 | 408790 | 470007 |
| Gphn | 4.0 | 0.8 | 4.8 | 1.7E-06 | 9.9E-03 | 5308 | 8848 | 96692 | 124140 |
| Rnu11 | 1.5 | 0.3 | 4.6 | 4.0E-06 | 1.6E-02 | 33 | 50 | 138 | 101 |
| Gm28872 | 1.5 | 0.3 | 4.5 | 5.5E-06 | 1.9E-02 | 34 | 50 | 138 | 101 |
| Gm44511 | 1.6 | 0.4 | 4.5 | 7.4E-06 | 2.2E-02 | 34 | 29 | 79 | 117 |
| CT010467.1 | 5.0 | 1.2 | 4.3 | 1.7E-05 | 4.6E-02 | 75034 | 161506 | 4055171 | 3369650 |
| Gm39383 | 3.3 | 0.8 | 4.3 | 2.0E-05 | 4.9E-02 | 5 | 5 | 28 | 82 |

Table S4. RNA-seq DEGs.

Differentially expressed genes (DEGs) determined by RNA-seq analysis of left ventricular tissue. Transcript counts are shown in the last four columns.
